# Autotaxin-Scavenging Nanoliposomes for Prolonged Colon Retention and Autophagy-Mediated Mucosal Immune Restoration in Colitis

**DOI:** 10.34133/bmr.0345

**Published:** 2026-03-19

**Authors:** So Won Jeon, Jun Kwon, Hee Gyeong Ko, Jong Sang Yoon, Hee Su Sohn, Jeong-Kee Yoon, Suk Ho Bhang, Min-Ho Kang, Han Young Kim

**Affiliations:** ^1^ Department of Biomedical-Chemical Engineering, The Catholic University of Korea, Bucheon 14662, Republic of Korea.; ^2^ Department of Biotechnology, The Catholic University of Korea, Bucheon 14662, Republic of Korea.; ^3^ Division of Pulmonary and Critical Care Medicine, Department of Medicine, Brigham and Women’s Hospital, Harvard Medical School, Boston, MA 02115, USA.; ^4^ Department of Systems Biotechnology, Chung-Ang University, Anseong 17546, Republic of Korea.; ^5^ School of Chemical Engineering, Sungkyunkwan University, Suwon 16419, Republic of Korea.; ^6^ Research Institute for Controlled Biomaterials of Regulated Cell Death, The Catholic University of Korea, Bucheon 14662, Republic of Korea.

## Abstract

Inflammatory bowel disease (IBD) is an immune-mediated disorder driven by overactivation of autotaxin (ATX), which elevates lysophosphatidic acid (LPA) signaling and suppresses autophagy, exacerbating intestinal inflammation. Given the pivotal role of autophagy in maintaining intestinal homeostasis, inhibiting ATX offers a dual therapeutic mechanism by both restoring autophagic activity and attenuating LPA-mediated inflammatory responses. Current treatments are hindered by nonspecific immunosuppression and frequent systemic side effects, underscoring the need for targeted, multifunctional therapeutic strategies. Here, we present a dual-functional nanotherapeutic platform, ATX-scavenging liposomes loaded with rapamycin (AS-Lipo@R), engineered for the oral treatment of acute colitis. Our proposed formulation incorporates BMP-22, a lipid ATX inhibitor that simultaneously functions as a structural building block of the liposomal membrane. Rapamycin, an autophagy activator, is encapsulated within the bilayer of liposomes. We confirmed that AS-Lipo@R exhibits strong binding affinity to extracellular ATX and mediates its lysosomal degradation upon cellular internalization, thereby demonstrating its ATX-scavenging property. In vitro, AS-Lipo@R inhibited inflammatory macrophage activation, promoted M2 macrophage polarization, and substantially restored autophagic activity in LPS/IFN-γ-stimulated macrophages. In vivo, oral administration of AS-Lipo@R led to preferential accumulation in ATX-overexpressing inflamed colonic tissue, resulting in reduced pro-inflammatory cytokine production, recovered autophagy, and enhanced intestinal barrier integrity in colitis mice. These findings highlight AS-Lipo@R as a synergistic and targeted nanomedicine that simultaneously modulates ATX and autophagy pathways, offering novel insights into immunomodulatory strategies for IBD treatment.

## Introduction

Inflammatory bowel disease (IBD), including Crohn’s disease and ulcerative colitis, is a chronic and relapsing inflammatory disorder of the gastrointestinal (GI) tract, primarily affecting the colon and rectal mucosa [1,2]. Over the past 2 decades, the global incidence of IBD has increased markedly not only in Western countries but also in Eastern populations, highlighting its growing worldwide health burden [3]. The pathogenesis of IBD is multifactorial and involves complex interactions among genetic susceptibility, environmental triggers, intestinal microbiota dysbiosis, and immune dysregulation [4,5]. Although a broad range of therapies, including dietary interventions, antibiotics, immunosuppressants, and surgery, are currently used to manage disease activity, these approaches often provide incomplete remission and may be associated with substantial long-term adverse effects. Therefore, there remains a critical unmet need for therapeutic strategies that more directly target disease-driving molecular pathways.

Recent evidence has identified autotaxin (ATX), a secreted lysophospholipase D belonging to the nucleotide pyrophosphatase/phosphodiesterase family, as an important mediator of intestinal inflammation [6–8]. ATX expression is markedly elevated in inflamed intestinal tissues and contributes to pathological immune activation by promoting lymphocyte recruitment and macrophage-driven inflammatory responses in the gut mucosa [9,10]. Mechanistically, ATX catalyzes the hydrolysis of lysophosphatidylcholine (LPC) to produce lysophosphatidic acid (LPA), a potent extracellular lipid mediator [11]. Importantly, the pathological relevance of ATX is primarily attributed to its enzymatic generation of LPA, rather than ATX itself functioning as a signaling molecule [12]. LPA activates LPA receptors (LPARs) expressed on immune and epithelial cells, thereby triggering multiple downstream inflammatory pathways, including AKT–mechanistic target of rapamycin (mTOR), RAS–mitogen-activated protein kinase (MAPK), and nuclear factor-κB (NF-κB) signaling [13,14]. Activation of these cascades collectively exacerbates intestinal inflammation by enhancing immune cell recruitment and cytokine production, impairing epithelial barrier integrity, and suppressing autophagy, ultimately sustaining mucosal injury and disease progression [15,16].

Autophagy is a conserved cellular homeostatic process that mediates the degradation and recycling of damaged organelles and macromolecules and is increasingly recognized as a key regulator of intestinal immune balance [17,18]. In the intestinal microenvironment, autophagy contributes to mucosal homeostasis by modulating macrophage polarization, cytokine secretion, and epithelial barrier maintenance [19,20]. Dysregulated autophagic flux has been implicated in IBD pathogenesis, where impaired autophagy promotes persistent inflammation and defective tissue repair [21]. Notably, excessive ATX/LPA signaling and autophagy inhibition frequently coexist in inflamed intestinal tissues, suggesting a pathological interplay that reinforces inflammatory progression [22].

**Fig. 1.**
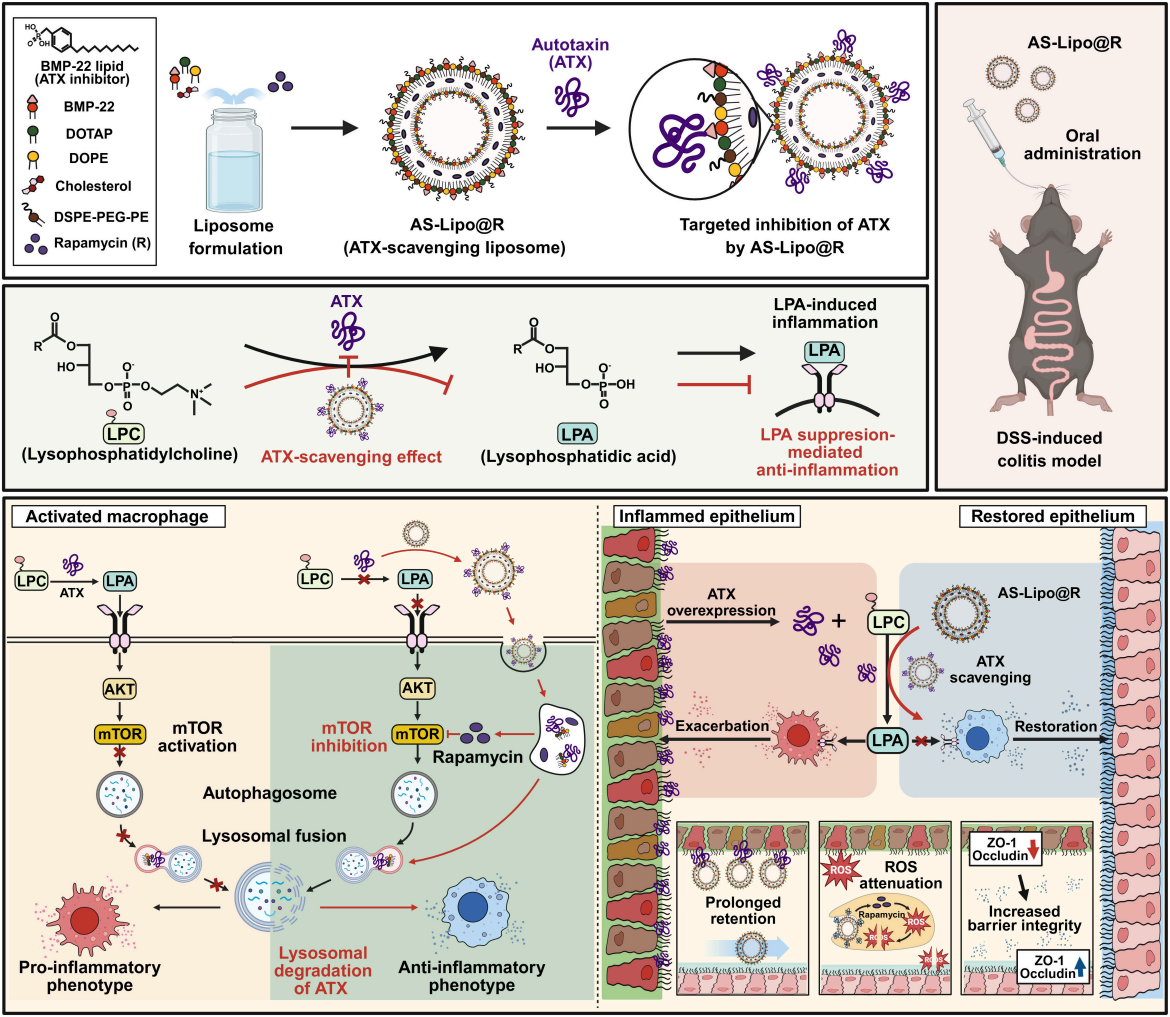
Schematic illustration of the therapeutic mechanism of AS-Lipo@R for colitis treatment. Autotaxin (ATX) is targeted and scavenged by AS-Lipo@R, which concurrently delivers rapamycin to enhance autophagy. AS-Lipo@R binds extracellular ATX and traffics to lysosomes, indicating lysosomal degradation as a cellular mechanism underlying ATX scavenging. By reducing lysophosphatidic acid (LPA) production and suppressing mTOR signaling, AS-Lipo@R drives macrophages toward an anti-inflammatory phenotype. After oral administration, AS-Lipo@R exhibits prolonged retention within the inflamed colon and accumulates at ATX-enriched sites. In the DSS-induced colitis model, AS-Lipo@R reduces ROS and inflammatory cytokines, restores tight junction proteins, improves epithelial barrier integrity, and supports overall mucosal recovery.

Based on this mechanistic framework, we reasoned that effective IBD intervention may require both suppression of ATX-mediated LPA production and restoration of autophagic activity [23]. To achieve this, we employed BMP-22, a bis(monoacylglycerol)phosphate-derived lipid inhibitor that directly binds the catalytic domain of ATX and blocks LPC-to-LPA conversion [24]. Despite its potent inhibitory activity, BMP-22 is limited by poor aqueous solubility and unfavorable pharmacokinetics, restricting its practical therapeutic application in free form [25]. To overcome these limitations and to enable inflammation-selective retention, we incorporated BMP-22 as a structural component of the liposomal bilayer, generating ATX-scavenging liposomes (AS-Lipo) designed to bind and sequester ATX within the inflamed intestinal microenvironment. In parallel, rapamycin, which acts as both an mTOR inhibitor and an autophagy activator [26], was encapsulated within the liposomal bilayer to generate the final dual-functional formulation (AS-Lipo@R). This platform was designed to integrate extracellular blockade of ATX-mediated LPA generation with intracellular restoration of autophagy, thereby reestablishing immune and metabolic homeostasis at both extracellular and intracellular levels (Fig. 1). In vivo, oral administration of AS-Lipo@R enabled preferential accumulation in ATX-overexpressing inflamed colonic tissue, leading to attenuation of intestinal inflammation through macrophage repolarization, restoration of autophagy, and reinforcement of intestinal barrier integrity. Our findings highlight the therapeutic potential of this combined immunomodulatory approach, offering a novel strategy for the treatment of inflammatory disorders.

## Methods

### Preparation of AS-Lipo@R

In this study, liposomes without BMP-22 (Lipo) and ATX-scavenging liposomes containing BMP-22 (AS-Lipo) were prepared using a standard thin-film hydration method. 1,2-Dioleoyl-3-trimethylammoniumpropane (DOTAP), 1,2-dioleoyl-sn-glycero-3-phosphoethanolamine (DOPE), and 1,2-distearoyl-sn-glycero-3-phosphoethanolamine-N-[methoxy(polyethylene glycol)-2000] (DSPE-PEG_2000_-PE) were dissolved in chloroform, whereas cholesterol and rapamycin were dissolved in 100% ethanol. BMP-22, 4-pentadecylbenzylphosphonic acid, was solubilized in dimethylformamide. All lipids were purchased from Avanti Polar Lipids (AL, USA). To prepare Lipo, the lipid components DOTAP, DOPE, cholesterol, and DSPE-PEG_2000_-PE were mixed in a molar ratio of 0.465:0.343:0.122:0.049. For AS-Lipo, BMP-22 was added at 2 mol % of the total lipid composition, with the other lipid components (DOTAP, DOPE, cholesterol, and DSPE-PEG_2000_-PE) maintaining their original molar ratio of 0.465:0.343:0.122:0.049 to account for the remaining molar percentage. The organic solvents were removed under a gentle nitrogen stream, and the resulting lipid film was further dried in a desiccator for 1 h. To synthesize AS-Lipo@R, 50 μg of rapamycin (Sigma-Aldrich, MO, USA) was incorporated into the lipid mixture before hydration. The resulting lipid film was hydrated with phosphate-buffered saline (PBS) at 37 °C for 1 h at 800 rpm. The resulting liposomal colloid was sequentially extruded through polycarbonate membrane filters with 1 μm, 400 nm, and 200 nm to produce uniformly sized particles.

### Physicochemical characterization of liposomes

The morphology of Lipo@R and AS-Lipo@R was characterized using cryogenic transmission electron microscopy (cryo-TEM). For sample preparation, 10 μl of the liposome suspension was applied onto a glow-discharged, holey carbon-coated copper grid under controlled conditions (15 °C, 100% humidity) using a Vitrobot Mark IV chamber (Thermo Fisher, USA). The vitrified samples were then transferred to a cryo-TEM holder (Model 626 single-tilt cryo-EM holder, USA) and maintained at approximately −180 °C during imaging. Cryo-TEM micrographs were acquired using a JEM-2100F electron microscope (JEOL, Japan). The particle size and zeta potential of Lipo@R and AS-Lipo@R were determined by dynamic light scattering (DLS) using a Zetasizer Nano ZS (Malvern Panalytical, UK). Colloidal stability was assessed by monitoring changes in particle size over 3 d by DLS. The encapsulation efficiency and release profile of rapamycin in AS-Lipo@R were analyzed using high-performance liquid chromatography (HPLC). For sample preparation, 1 ml of 0.1% Triton X-100 was added to the AS-Lipo@R suspension and incubated at 37 °C for 10 min. The HPLC column temperature was maintained at 60 °C, with a mobile phase consisting of 10 mM ammonium acetate and acetonitrile (25:75, v/v). The flow rate was set to 1.5 ml/min, and 10 μl of each sample was injected for analysis. The in vitro release of rapamycin from AS-Lipo@R was evaluated using a dialysis method under sink conditions with a 3.5-kDa molecular-weight-cutoff membrane. Samples were collected over 3 d and quantified by HPLC. The encapsulation efficiency of rapamycin was calculated as the percentage of rapamycin encapsulated in AS-Lipo@R relative to the total amount initially added, according to the equation = (encapsulated rapamycin/total rapamycin added) × 100 (%). The loading capacity of rapamycin was calculated as the molar fraction of rapamycin relative to the total lipid molar amount, according to the equation = (encapsulated rapamycin/total mol of lipids) × 100 (mol %).

### AS-Lipo@R stability and release behavior in simulated GI conditions

The GI stability of AS-Lipo@R was evaluated under simulated gastric and intestinal environments. Simulated gastric fluid (SGF) and simulated intestinal fluid (SIF) were commercially purchased and prepared according to U.S. Pharmacopeia (USP) specifications. AS-Lipo@R was diluted in SGF or SIF at a volume ratio of 1:3 (formulation:simulated fluid) and incubated at 37 °C with agitation at 100 rpm. The incubation periods were set to 2 h in SGF and 6 h in SIF to reflect physiologically relevant gastric and intestinal transit times. At predetermined time points, aliquots were collected, and the hydrodynamic particle size and polydispersity index (PDI) were measured using DLS to assess colloidal stability and structural integrity. Rapamycin release from AS-Lipo@R was evaluated using SGF and SIF in a dialysis-based release system. AS-Lipo@R was mixed with SGF or SIF at a 1:3 volume ratio and transferred into a dialysis bag (molecular weight cutoff, 3.5 kDa). The sealed bag was immersed in 50 ml of the corresponding simulated digestive fluid and incubated at 37 °C with agitation at 100 rpm. At predetermined intervals, 1 ml of sample was collected from the receptor medium and analyzed by HPLC to quantify released rapamycin.

### Evaluation of ATX binding and inhibitory activity

ATX inhibitory activity was first quantified using a choline release assay based on the Amplex Red method. The reaction buffer consisted of 250 mM NaCl, 125 mM magnesium acetate, 25 mM Tris (pH 8.2), and 0.2% bovine serum albumin (BSA) (all from Sigma-Aldrich, USA). Free BMP-22, Lipo, or AS-Lipo was preincubated with an enzymatic reaction mixture containing 1 nM recombinant mouse ENPP2/ATX protein (R&D Systems, MN, USA) and 30 μM 16:0 LPC (1-palmitoyl-2-hydroxy-sn-glycero-3-phosphocholine) at 37 °C for 1 h with gentle agitation. Subsequently, a detection cocktail containing 10 μM Amplex Red (Thermo Fisher, USA), 2 U/ml horseradish peroxidase (HRP), and 1 U/ml choline oxidase was added. Fluorescence intensity was measured at 540 nm (excitation) and 590 nm (emission) using a GloMax microplate reader (Promega, USA). To further quantify the binding capacity of AS-Lipo toward ATX, an ENPP2/Autotaxin ELISA kit (Assay Genie, Ireland) was used. Recombinant mouse ENPP2/ATX protein was incubated with Lipo or AS-Lipo at 37 °C for 1 h to facilitate binding. After incubation, the mixtures were centrifuged at 12,000*g* for 10 min using a 300-kDa molecular-weight-cutoff Vivaspin centrifugal filter unit (Sartorius, USA) to separate liposome-bound ATX from unbound ATX. The retained fraction representing ATX associated with each liposomal formulation was analyzed by enzyme-linked immunosorbent assay (ELISA) according to the manufacturer’s protocol.

### Cellular uptake and lysosomal trafficking of liposomes in macrophages

RAW 264.7 murine macrophages were cultured in Dulbecco’s modified Eagle’s medium (DMEM; Gibco, USA) supplemented with 10% (v/v) fetal bovine serum (FBS; Gibco, USA) and 1% (v/v) penicillin–streptomycin (Gibco, USA) at 37 °C in a humidified atmosphere containing 5% CO₂. For cytotoxicity assessment, cells were seeded in 96-well plates at a density of 5 × 10^3^ cells per well and treated with various concentrations of AS-Lipo@R for 24, 48, or 72 h. Cell viability was determined using a CCK-8 assay kit (EZ-Cytox, DoGenBio, Korea) according to the manufacturer’s protocol. For macrophage polarization studies, cells were seeded in 6-well plates at a density of 1 × 10^6^ cells per well, allowed to adhere overnight, and then stimulated with lipopolysaccharide (LPS; 100 ng/ml) and interferon-γ (IFN-γ; 20 ng/ml) in the presence of Lipo@R (10 to 285 ng/ml) or AS-Lipo@R (0.28 to 285 ng/ml) for 24 h.

For cellular uptake and lysosomal trafficking analysis, Lipo or AS-Lipo was labeled with DiOC18(3); 3,3′-dioctadecyloxacarbocyanine perchlorate (DiO; Thermo Fisher Scientific, USA) by incorporating DiO-lipid at a molar ratio of 0.1:1 (DiO:lipid). Next, recombinant mouse ENPP2/ATX protein was thiolated using a 20-fold molar excess of Traut’s reagent for 1 h at room temperature and subsequently conjugated to Alexa Fluor 647 maleimide overnight at 4 °C in the dark. Unreacted fluorophore was removed using a Zeba Spin Desalting Column (Thermo Fisher Scientific, USA). Alexa Fluor 647-labeled ATX was incubated with DiO-stained Lipo or AS-Lipo at 37 °C for 1 h, and unbound ATX was removed using a 300-kDa molecular-weight-cutoff Vivaspin centrifugal filter (Sartorius, USA). RAW 264.7 macrophages were treated with ATX-bound AS-Lipo and subsequently stained with 60 nM LysoTracker (Thermo Fisher Scientific, USA) for 6 h to visualize lysosomes. The colocalization of ATX and liposomal formulations with lysosomes was then analyzed by fluorescence microscopy.

### Analysis of macrophage polarization and inflammatory responses

In vitro immunological analyses were conducted after establishing an inflammatory environment mimicking IBD by stimulating cells with LPS and IFN-γ, followed by treatment with Lipo@R or AS-Lipo@R. Following treatment, cells were harvested and subjected to immunophenotypic analysis. M1 polarization was assessed using phycoerythrin–anti-CD86 (BioLegend, 1005007) and allophycocyanin–anti-CD80 (BioLegend, 104716). Cells were incubated with fluorescent-conjugated antibodies at 4 °C for 30 min in the dark. The stained cells were analyzed using a CytoFLEX flow cytometer (Beckman Coulter, USA), and data acquisition and analysis were performed with FlowJo software (Tree Star, USA). Western blot analysis was performed to confirm the signaling pathways involved in macrophage activation. Cell pellets were dissolved in cold radioimmunoprecipitation assay lysis buffer containing a protease inhibitor cocktail. After centrifugation at 13,000rpm for 20 min, supernatants were collected, and protein concentrations were quantified by Bradford assay. Subsequently, sodium dodecyl sulfate–polyacrylamide gel electrophoresis (SDS-PAGE) was run under standard electrophoresis conditions, with 20 μg of total protein loaded in each lane. Separated proteins were transferred onto 0.45-μm polyvinylidene difluoride (PVDF) membranes at 70 V for 90 min and blocked with 3% skim milk powder at room temperature for 1 h. Membranes were then incubated with primary antibodies at 4 °C overnight (ATX (1:1,000, ab77104, Abcam, USA), mTOR (1:3,000, A25581), p-mTOR (1:1,000, AP0115), AKT (1:1,000, A18120), p-AKT (1:1,000, AP1208), p62 (1:20,000, AP19700), LC3 (1:10,000, A27200PM), all from ABclonal, USA, and β-actin (1:1,000, 8H10D10, Cell Signaling Technology, USA)). The HRP-conjugated secondary antibodies were subsequently added and incubated at room temperature for 1 h. Finally, protein bands were visualized using enhanced chemiluminescence reagent and imaged with a ChemiDoc imaging system (Bio-Rad, USA). Band intensities relative to the control β-actin were quantified using ImageJ software. For cytokine secretion analysis, culture supernatants were collected and analyzed by ELISA kits (R&D Systems, USA) according to the manufacturer’s instructions. To evaluate the expression levels of inflammation and autophagy-related genes, total RNA was extracted from the cells using TRIzol reagent (Thermo Scientific, USA) and transcribed into cDNA using AccuPower RT PreMix & Master Mix (Bioneer, Korea). The cDNA was subjected to quantitative real-time polymerase chain reaction (qRT-PCR) using an SYBR Green RT-qPCR kit (Enzynomics, Korea) on an ABI StepOne Real-Time PCR System (Applied Biosystems, USA). The primers used for qRT-PCR are listed in Table S1. The mRNA expression of various genes was referenced against β-actin. Related gene expression was calculated against β-actin using the 2^−ΔΔCT^ method.

### Mouse model of DSS-induced colitis

All animal experimental procedures were performed according to the guidelines and with the approval of the Institutional Animal Care and Use Committee (IACUC) of The Catholic University of Korea (approval no.: CUK-IACUC-2023-010). Female C57BL/6 mice (6 to 8 weeks old) were purchased from Koatech (Korea) and housed under standard conditions with a 12-h light/dark cycle and ad libitum access to food and water. IBD was induced by administering 3% dextran sulfate sodium (DSS) (MP Biomedicals, USA) in their drinking water for 6 consecutive days. This was followed by a 4-d recovery period with regular DSS-free water. Mice were randomly divided into untreated and saline-, Free R-, AS-Lipo-, Lipo@R-, and AS-Lipo@R-treated groups. Treatments were administered orally every day for a total of 5 doses, starting on day 2, with an injection volume of 200 μl per dose. For delayed-treatment experiment, acute colitis was induced by administration of 3% DSS for 5 consecutive days, followed by replacement with normal water. Treatments were orally administered once daily during the recovery phase (days 5 to 9), and therapeutic outcomes were evaluated on day 10. Mice were monitored daily for changes in body weight, food intake, stool consistency, and rectal bleeding. Body weight was recorded on day 0 (before DSS administration) and immediately before sacrifice. Disease progression was evaluated using the disease activity index (DAI), which combines scores for body weight loss, stool consistency, and bleeding [27].

### In vivo imaging of GI and colonic biodistribution

To evaluate the in vivo localization of liposomes, Lipo and AS-Lipo formulations were fluorescently labeled with DiD (1,1′-dioctadecyl-3,3,3′,3′-tetramethylindodicarbocyanine, 4-chlorobenzenesulfonate salt; V22887, Thermo Fisher Scientific, USA) and administered orally to mice. Fluorescence signals in the GI tract were captured at predetermined time points (2, 4, 8, and 24 h) using a FOBI imaging system (NeoScience, Korea). At 24 h post-administration, mice were euthanized, and the colon, along with major organs, including the lung, liver, heart, spleen, kidney, and bladder, was harvested for ex vivo fluorescence imaging. To evaluate ATX-mediated nanoparticle capture in the colon of IBD mice, frozen colon sections were stained with ATX and F4/80. Fluorescence images were acquired using a fluorescence microscope, and fluorescence intensity was quantified using image analysis software.

### Histology and immunohistochemistry

Colon and major organs were collected from mice and fixed in 4% paraformaldehyde (PFA) for 24 h at room temperature. The tissues were cryoprotected sequentially in 15% sucrose solution overnight and then in 30% sucrose solution for at least 6 h. After cryoprotection, the samples were embedded in optimal cutting temperature (OCT) compound and frozen at −80 °C. Frozen tissues were cut into 7-μm-thick sections and placed on glass slides. Sections were stained with a hematoxylin and eosin kit (ab245880, Abcam, USA) and mounted with synthetic resin. The immune cells within tissues were detected by the immunohistochemical staining method. Briefly, frozen tissue sections were thawed and washed twice with PBS for 5 min each, followed by incubation in a blocking solution containing 90% PBS, 10% goat serum, and 0.6% Triton X-100 (X-100, Sigma-Aldrich, USA) for 2 h at room temperature. After blocking, the sections were incubated with primary antibodies overnight at 4 °C in a humidified chamber. To detect M1 and M2 macrophages, the following primary antibodies were used: fluorescein isothiocyanate-conjugated anti-mouse F4/80 (123107, BioLegend, USA), Alexa Fluor 594–anti-mouse CD80 (104753, BioLegend, USA), Alexa Fluor 594–anti-mouse inducible nitric oxide synthase (iNOS) (696803, BioLegend, USA), Alexa Fluor 488–anti-mouse Arg-1 (53369780, Invitrogen, USA), and Alexa Fluor 647–anti-mouse CD163 (155329, BioLegend, USA). Finally, to assess the integrity of the GI tract epithelium, tissue sections were stained with Alexa Fluor 488-conjugated anti-mouse Occludin (133256, Santa Cruz, USA) and Alexa Fluor 647–anti-mouse ZO-1 (MA339100, Invitrogen, USA).

### Statistical analysis

GraphPad Prism was used for statistical analysis. All data are presented as mean ± SD. Statistical significance was determined by 1-way or 2-way analysis of variance (ANOVA) followed by Tukey’s post hoc test. *P < 0.05, **P < 0.01, ***P < 0.001, and ****P < 0.0001. ns, not significant.

## Results

### Characterization and ATX-scavenging activity of AS-Lipo@R

**Fig. 2.**
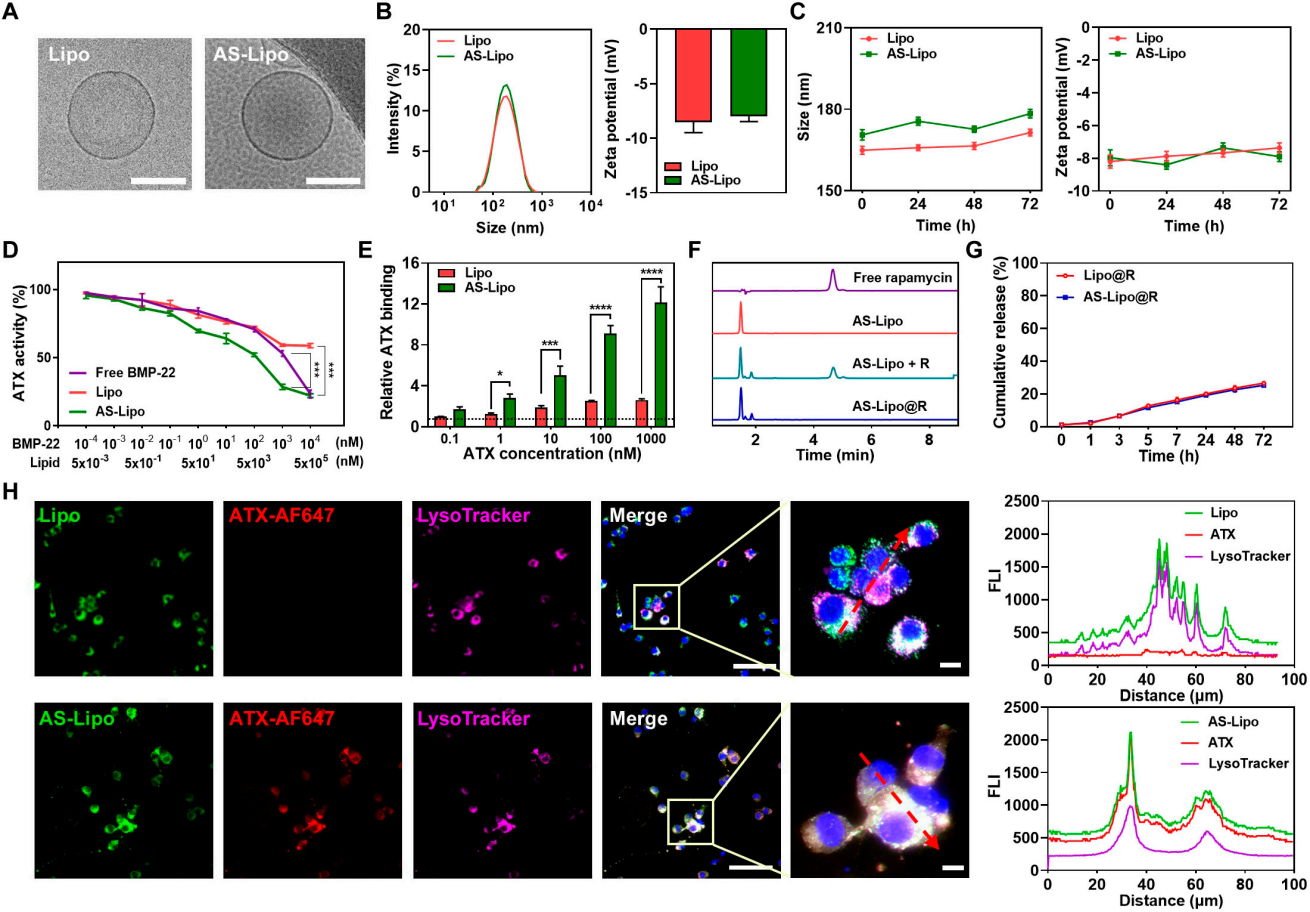
Characterization and ATX-scavenging activity of AS-Lipo@R. (A) Cryo-TEM images of Lipo and AS-Lipo (scale bar, 100 nm). (B) Particle size and zeta potential of Lipo and AS-Lipo measured by DLS. (C) Colloidal stability of Lipo and AS-Lipo was evaluated by monitoring changes in particle size and zeta potential over 72 h in PBS using DLS. (D) Concentration-dependent inhibition of recombinant mouse ATX activity as a function of BMP-22 concentration in free BMP-22, Lipo, and AS-Lipo, evaluated using a choline release assay. (E) ATX binding to the surface of Lipo and AS-Lipo as a function of ATX concentration, quantified by ELISA. (F) HPLC chromatograms showing rapamycin (R) encapsulation in AS-Lipo@R, as indicated by the disappearance of the free R peak. (G) Cumulative release profiles of rapamycin from Lipo@R and AS-Lipo@R in PBS over 72 h measured by HPLC. (H) Fluorescence imaging showing colocalization of DiO-labeled Lipo or AS-Lipo (green), Alexa Fluor 647 (AF647)-labeled ATX (red), and LysoTracker (purple) in RAW 264.7 macrophages. AS-Lipo-bound ATX is internalized and colocalizes with lysosomes, indicating lysosomal degradation. Right panels show fluorescence intensity profiles along the indicated lines, quantifying the colocalization (scale bar, 100 and 50 μm). Statistical significance was determined by one-way ANOVA with Tukey’s post hoc test (*n* = 3). *P < 0.05, **P < 0.01, ***P < 0.001, and ****P < 0.0001.

To endow liposomes with both ATX-inhibiting and autophagy-activating functions, we constructed a dual-functional liposomal system in which BMP-22 and rapamycin were co-assembled within the lipid bilayer. BMP-22, a lipid-based ATX inhibitor, was integrated into the membrane as a structural component, while rapamycin was encapsulated within the bilayer to enable synergistic modulation of ATX and autophagy. Cryo-TEM analysis revealed that AS-Lipo exhibited a uniform spherical morphology without aggregation, comparable to Lipo indicating preserved structural integrity after BMP-22 incorporation (Fig. 2A). DLS analysis showed average particle sizes of 156.4 ± 4.9 nm (Lipo) and 166.4 ± 2.7 nm (AS-Lipo), with zeta potentials of −8.0 mV and −7.2 mV, respectively (Fig. 2B). Importantly, both formulations exhibited similarly low PDI values, 0.125 for Lipo and 0.134 for AS-Lipo, indicating a narrow size distribution and high colloidal uniformity (Fig. S1). Furthermore, both Lipo and AS-Lipo remained colloidal stable in PBS for up to 3 d, showing no significant changes in particle size and zeta potential during the observation period (Fig. 2C). Given that the GI tract exhibits a broad pH range from approximately pH 1 to 2 in the stomach to pH 8 in the large intestine, it is essential to evaluate whether AS-Lipo@R can retain its structural integrity under varying pH conditions during oral administration [28]. To assess the pH-dependent stability, AS-Lipo@R was first prepared in PBS at pH 7.4 and subsequently diluted into PBS buffers adjusted to pH values ranging from 1 to 7. Across this range, the particle size remained stable, with an average diameter of approximately 160 ± 20 nm (Fig. S2A). Similarly, the zeta potential was consistently maintained around −8 mV, indicating stable surface charge and colloidal dispersion without notable aggregation or degradation. Furthermore, AS-Lipo@R maintained its size and zeta potential value for up to 72 h under these varying pH conditions, confirming its durability in the GI environment (Fig. S2B). In addition to pH stability, we further evaluated GI stability under enzyme-containing, simulated GI conditions to better mimic oral transit. Specifically, Lipo, AS-Lipo, Lipo@R, and AS-Lipo@R were incubated in SGF and SIF, and their physicochemical properties were monitored over time. As shown in Fig. S3A, all formulations maintained stable hydrodynamic diameters without noticeable aggregation or collapse throughout incubation in PBS, SGF, and SIF for up to 16 h. Consistently, the PDI values remained low and did not show significant broadening under either SGF or SIF conditions (Fig. S3B), indicating preserved colloidal uniformity and structural integrity of the liposomal bilayer during simulated GI exposure. Next, to verify whether the ATX-inhibiting activity of BMP-22 was preserved after incorporation into AS-Lipo, ATX enzymatic activity was quantified using a choline release assay that measures LPC hydrolysis (Fig. 2D). Free BMP-22 was included as a positive control to validate ATX inhibition. As a result, AS-Lipo showed significantly stronger ATX inhibitory activity than Lipo across all concentrations, indicating that the lipid-based ATX inhibitor retained its functionality after bilayer incorporation. Interestingly, at equivalent BMP-22 concentrations (1 μM), AS-Lipo displayed stronger inhibition than free BMP-22, suggesting that membrane-embedded BMP-22 provides improved local interaction with ATX or enhanced stability in the lipid microenvironment. This enhanced inhibitory effect is also likely attributable to membrane-associated interfacial presentation and local enrichment at the lipid–water interface [29]. In contrast, Lipo showed a mild inhibitory effect only at the highest concentrations, which is likely due to nonspecific lipid–protein interactions rather than genuine enzymatic inhibition. To further validate the ATX-binding capability, an ELISA-based binding assay was performed (Fig. 2E). Consistent with the enzymatic results, AS-Lipo demonstrated significantly stronger ATX binding than Lipo, confirming that BMP-22 maintains its specific ATX recognition and functional integrity when incorporated into the liposomal formulation. To confirm the encapsulation of rapamycin within the liposomal bilayer, HPLC analysis was performed (Fig. 2F). The characteristic peak of free rapamycin was clearly detected in the sample containing physically mixed AS-Lipo and rapamycin (AS-Lipo + R), whereas the peak disappeared in AS-Lipo@R prepared via the thin-film hydration process, indicating complete encapsulation of rapamycin within the liposomal structure. The encapsulation efficiency was determined to be 64.9% based on the recovered rapamycin peak area relative to the initial input. The loading capacity, defined as the molar ratio of rapamycin to total lipids, was 3.5 mol %. The release profile of rapamycin was then examined to evaluate its stability (Fig. 2G). Both Lipo@R and AS-Lipo@R exhibited sustained release behavior, with less than 20% cumulative drug release observed over 72 h, which corresponds to the approximate transit time to the colon. To further evaluate drug release behavior during oral administration, rapamycin release profiles were additionally assessed under sequential GI conditions using SGF (pH 1.5) followed by SIF (pH 6.5) (Fig. S4). Under SGF conditions, both Lipo@R and AS-Lipo@R showed minimal premature rapamycin release, indicating that the liposomal bilayer effectively protected the encapsulated drug from rapid leakage in the acidic gastric environment. After transition to SIF conditions, a gradual and sustained increase in cumulative release was observed over time, reaching approximately 25% at 72 h. Next, to investigate the intracellular fate of liposomal formulations following ATX interaction, DiO-labeled Lipo or AS-Lipo was pre-incubated with Alexa Fluor 647-conjugated ATX and purified to remove unbound ATX. The resulting ATX-bound liposomes were then applied to RAW 264.7 macrophages, and cellular localization was visualized by fluorescence microscopy using LysoTracker as a lysosomal marker (Fig. 2H). In cells treated with AS-Lipo, fluorescence signals from liposomes, ATX, and lysosomes were strongly colocalized, indicating that AS-Lipo forms stable complexes with extracellular ATX, which are subsequently internalized and trafficked to lysosomes for degradation. In contrast, Lipo treatment resulted in a clear overlap with LysoTracker but showed no detectable ATX signal, indicating that Lipo was nonspecifically internalized and degraded in lysosomes without interacting with extracellular ATX. These results provide direct evidence that BMP-22-integrated AS-Lipo specifically recognizes and sequesters extracellular ATX, mediating its endocytosis and subsequent lysosomal degradation, which represents a key mechanism underlying the ATX-scavenging function of the nanocarrier.

### ATX inhibition and autophagy restoration via AS-Lipo@R

**Fig. 3.**
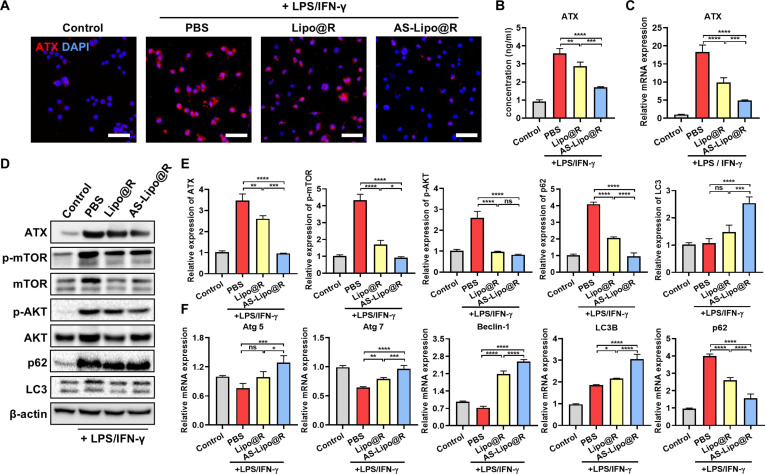
ATX inhibition and autophagy restoration of macrophages via AS-Lipo@R. (A) Representative immunofluorescence images of ATX (red) and nuclei [4′,6-diamidino-2-phenylindole (DAPI), blue] in RAW 264.7 cells stimulated with LPS/IFN-γ and treated with PBS, Lipo@R, or AS-Lipo@R (scale bar, 100 μm). (B) Quantification of ATX protein levels measured by ELISA (*n* = 4). (C) Relative ATX mRNA expression measured by qRT-PCR and calculated using the 2^−ΔΔCT^ method, normalized to β-actin (*n* = 4). (D) Western blot analysis of ATX, total and phosphorylated mTOR (mTOR, p-mTOR), total and phosphorylated AKT (AKT, p-AKT), and autophagy-related proteins (p62 and LC3) in LPS/IFN-γ-stimulated RAW 264.7 cells treated with PBS, Lipo@R, or AS-Lipo@R. β-Actin was used as a loading control. (E) Densitometric quantification of Western blot bands normalized to β-actin and expressed as relative protein levels (*n* = 3 independent biological replicates). (F) qRT-PCR analysis of autophagy-related genes (Atg5, Atg7, Beclin-1, LC3B, and p62) (*n* = 4). All data are presented as mean ± SD. Statistical significance was determined by one-way ANOVA followed by Tukey’s post hoc test. *P < 0.05, **P < 0.01, ***P < 0.001, and ****P < 0.0001. ns, not significant.

Given that ATX-mediated signaling suppresses autophagy and promotes inflammation, we investigated whether ATX inhibition could restore autophagy in activated macrophages. RAW 264.7 macrophages were cotreated with LPS/IFN-γ and liposomal formulations (Lipo@R or AS-Lipo@R). Pretreatment cytotoxicity assays revealed minimal toxicity for both formulations, and cell viability was comparable to that of free rapamycin at the same concentration (Fig. S5). Immunofluorescence staining revealed strong ATX expression in LPS/IFN-γ-activated macrophages (Fig. 3A), whereas Lipo@R and AS-Lipo@R reduced ATX fluorescence intensity to ~21% and ~12% of the LPS/IFN-γ group, respectively (Fig. S6). These results suggest that AS-Lipo binds extracellular ATX, facilitating its cellular internalization and thereby reducing its ability to activate inflammatory signaling pathways. To further verify the regulation of ATX expression, ELISA and qRT-PCR were performed to assess ATX protein levels and mRNA, respectively (Fig. 3B and C). Both analyses showed that LPS/IFN-γ stimulation markedly increased ATX expression at both the transcript and protein levels, confirming transcriptional activation under inflammatory conditions. AS-Lipo treatment moderately reduced ATX expression, whereas AS-Lipo@R led to a pronounced suppression of both mRNA and protein levels. To clarify downstream signaling, Western blot analysis was performed to assess ATX/AKT/mTOR activation and autophagy markers (Fig. 3D and E). LPS/IFN-γ increased AKT and mTOR phosphorylation accompanied by p62 accumulation and reduced LC3, indicating impaired autophagy [30]. In contrast, AS-Lipo@R decreased p62 and restored LC3, supporting recovery of autophagic turnover. qRT-PCR results were consistent with these findings: Inflammatory stimulation down-regulated Atg5, Atg7, Beclin-1, and LC3B while up-regulating p62, whereas AS-Lipo@R robustly reversed these transcriptional changes (Fig. 3F) [31,32]. To provide direct morphological evidence for autophagy restoration, we further performed immunocytochemical staining of LC3 and p62 in macrophages (Fig. S7). LPS/IFN-γ stimulation resulted in marked p62 puncta accumulation, accompanied by limited LC3 puncta formation, consistent with disrupted autophagic flux. In contrast, AS-Lipo@R significantly increased LC3 puncta per cell while reducing p62 puncta compared with PBS and Lipo@R groups, indicating enhanced autophagosome formation together with improved substrate clearance. Collectively, these results demonstrate that AS-Lipo@R suppresses ATX-driven AKT/mTOR signaling and restores autophagy under inflammatory conditions.

### Immunomodulatory effects of AS-Lipo@R in macrophage repolarization

**Fig. 4.**
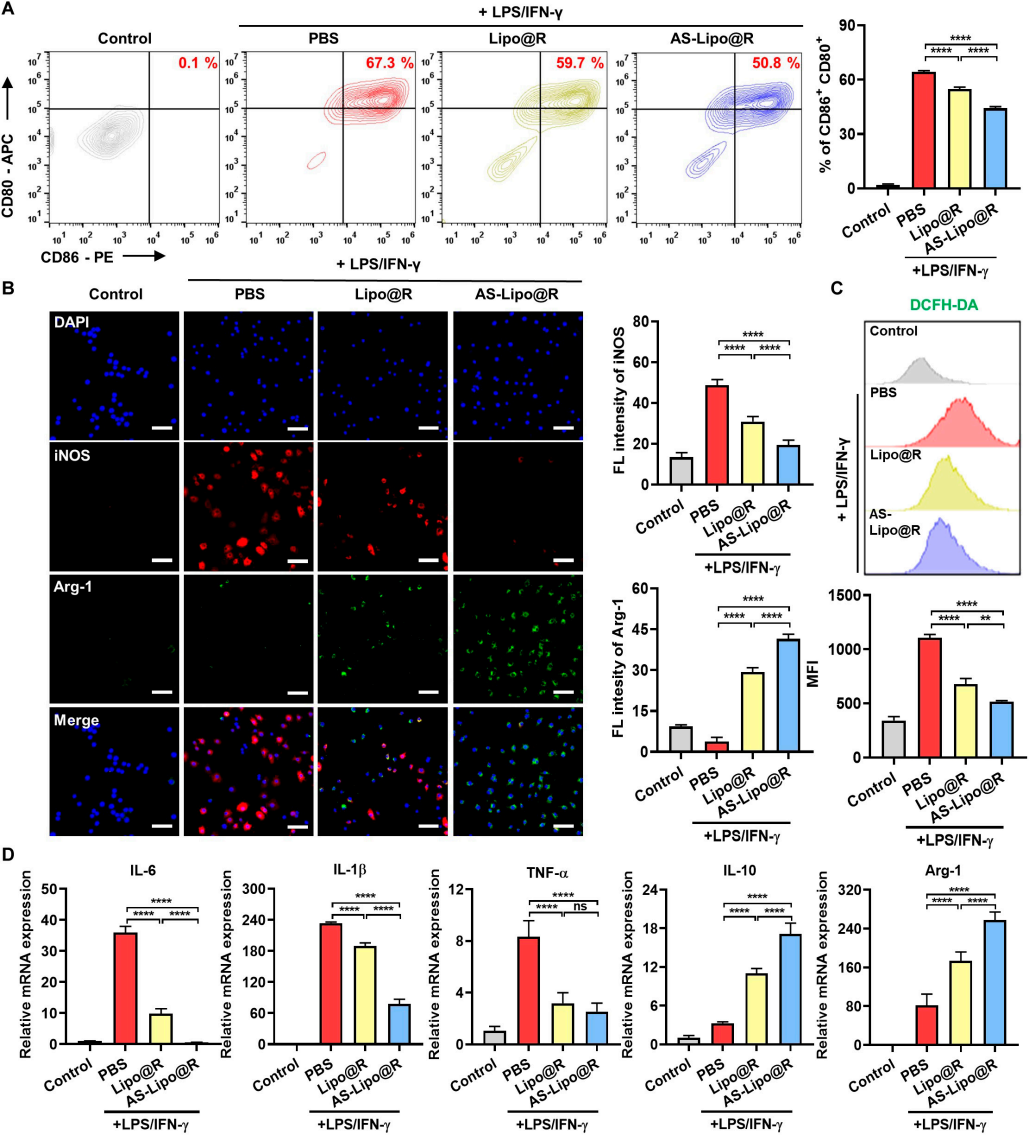
Immunomodulatory effects of AS-Lipo@R in macrophage repolarization. (A) Flow cytometric analysis of M1-type macrophages (CD80^+^CD86^+^) in RAW 264.7 cells stimulated with LPS/IFN-γ and treated with PBS, Lipo@R, or AS-Lipo@R. Representative scatterplots and quantification are shown (*n* = 3). (B) Immunofluorescence images and quantitative analysis showing macrophage polarization, demonstrating modulation of M1 (iNOS^+^, red) and M2 (Arg-1^+^, green) populations (scale bar, 50 μm; *n* = 3). (C) Intracellular ROS levels measured by DCFH-DA staining and flow cytometry in LPS/IFN-γ-stimulated RAW 264.7 cells treated with the indicated formulations. (D) mRNA expression of M1-associated genes (IL-6, IL-1β, and TNF-α) and M2-associated genes (IL-10 and Arg-1) measured by qRT-PCR (*n* = 4). Statistical significance was determined using one-way ANOVA with Tukey’s post hoc test. *P < 0.05, **P < 0.01, ***P < 0.001, and ****P < 0.0001. ns, not significant.

Aberrant macrophage polarization toward the M1 phenotype plays a critical role in IBD progression. Previous studies have shown that reprogramming pro-inflammatory M1 macrophages into the anti-inflammatory M2 phenotype contributes to mucosal healing and immune resolution [33]. To assess the immunomodulatory potential of AS-Lipo@R, we evaluated the expression of M1 surface markers CD80 and CD86 by flow cytometry. LPS/IFN-γ stimulation markedly increased the expressions of both markers, confirming induction of M1 activation. Treatment with Lipo@R and AS-Lipo@R reduced expression levels, and AS-Lipo@R further decreased M1 marker expression by approximately 20% compared with Lipo@R, indicating more effective suppression of macrophage polarization (Fig. 4A). In parallel, analysis of the M2 marker CD206 revealed that AS-Lipo@R significantly increased the proportion of CD206^+^ macrophages relative to Lipo@R and control groups (Fig. S8), demonstrating that attenuation of the M1 phenotype was accompanied by a shift toward an anti-inflammatory M2 state rather than simple inhibition of inflammatory signaling. To further evaluate macrophage polarization, immunocytochemistry was conducted to examine iNOS and Arg-1 expression (Fig. 4B). iNOS is a hallmark of pro-inflammatory M1 macrophages, whereas Arg-1 is a representative M2 marker involved in arginine metabolism [34]. LPS/IFN-γ treatment increased iNOS expression, whereas AS-Lipo reduced iNOS and increased Arg-1, suggesting partial repolarization. Notably, AS-Lipo@R reduced iNOS to near-control levels while markedly up-regulating Arg-1, indicating robust M2 polarization. Consistently, immunocytochemical staining for CD80 (M1) and CD163 (M2) further confirmed that AS-Lipo@R suppressed CD80 and enhanced CD163 expression (Fig. S9). Reactive oxygen species (ROS) sustain the inflammatory microenvironment and promote M1 polarization [35]. Flow cytometry and microscopic analysis using 2′,7′-dichlorodihydrofluorescein diacetate (DCFH-DA) demonstrated that LPS/IFN-γ stimulation markedly increased intracellular ROS levels, whereas both Lipo@R and AS-Lipo@R significantly suppressed ROS accumulation, with AS-Lipo@R showing the strongest reduction (Fig. 4C and Fig. S10). To further evaluate anti-inflammatory effects, macrophage-associated cytokines were quantified using qRT-PCR (Fig. 4D) and ELISA (Fig. S11). LPS/IFN-γ markedly up-regulated tumor necrosis factor-α (TNF-α), interleukin-1β (IL-1β), and IL-6, while AS-Lipo@R significantly suppressed these cytokines at both mRNA and protein levels. In parallel, IL-10 expression and secretion were increased, supporting an M2-like phenotypic shift.

### Prolonged retention of AS-Lipo in ATX-overexpressing inflamed colon

**Fig. 5.**
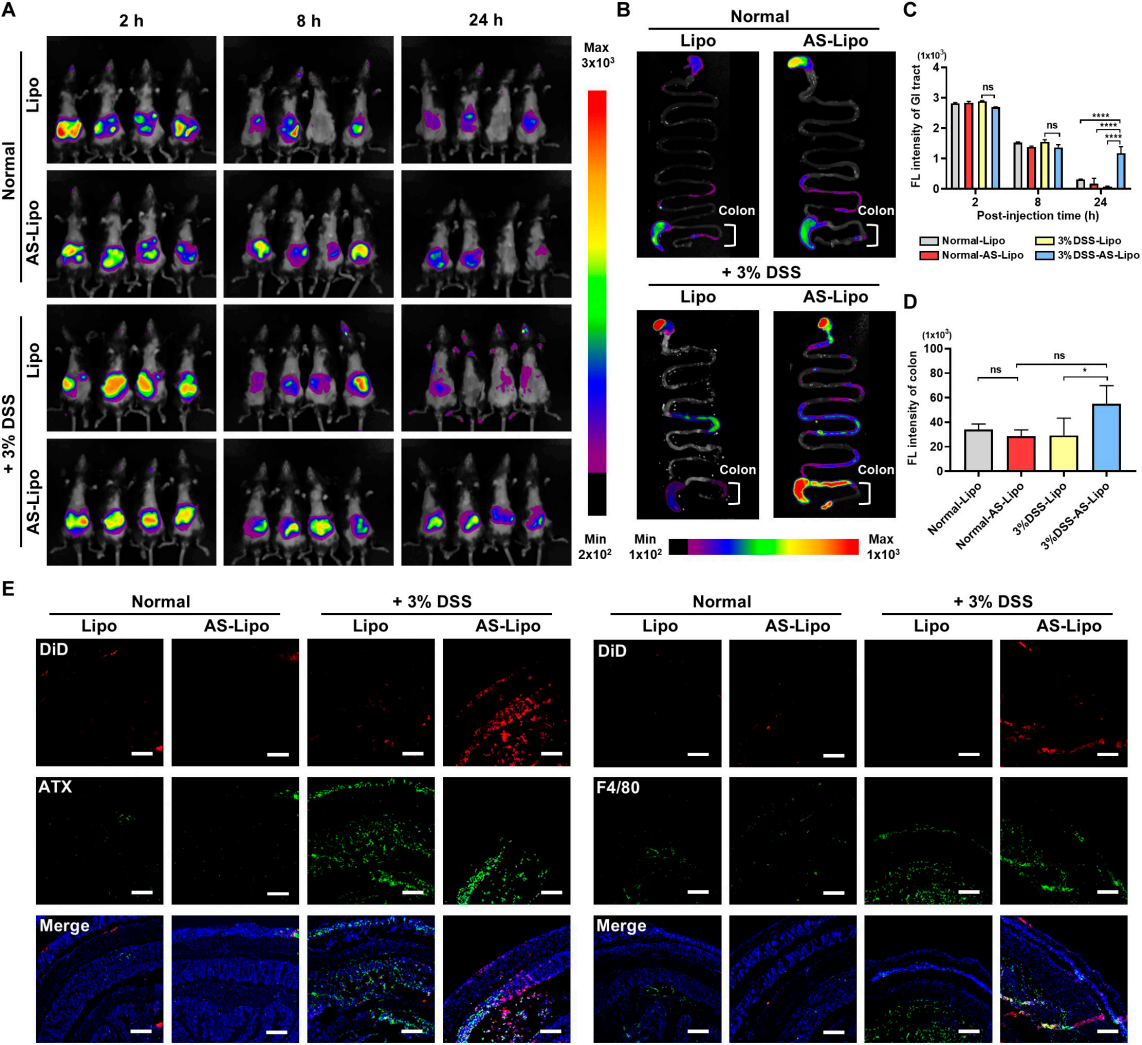
Prolonged retention of AS-Lipo in ATX-overexpressing inflamed colon. DiD-labeled Lipo and AS-Lipo were orally administered to normal mice and 3% DSS-treated mice. (A) In vivo fluorescence imaging showing time-dependent biodistribution and abdominal accumulation of DiD-labeled Lipo and AS-Lipo in normal mice and 3% DSS-treated mice at 2, 8, and 24 h after oral administration (*n* = 4). Fluorescence images were acquired using an IVIS imaging system under identical acquisition settings across all groups. (B) Ex vivo gastrointestinal (GI) imaging at 24 h post-administration displaying colon-specific accumulation of DiD-labeled Lipo and AS-Lipo in normal and 3% DSS-treated mice. The colon regions are indicated by brackets. (*n* = 4). (C) Quantification of fluorescence intensity from the entire GI tract (*n* = 4). (D) Quantification of colon-specific fluorescence intensity at 24 h, extracted from the colon regions indicated in the ex vivo GI tract images. (E) Immunofluorescence staining of colon tissue sections showing localization of DiD-labeled liposomes (red) with ATX (green, left panels) or macrophage marker F4/80 (green, right panels) in normal and 3% DSS-treated mice. Nuclei are counterstained with DAPI (blue). Scale bar, 100 μm. Statistical analysis was performed by one-way ANOVA with Tukey’s post hoc test. *P < 0.05, **P < 0.01, ***P < 0.001, and ****P < 0.0001. ns, not significant.

To assess the in vivo targeting and retention capability of the liposomal formulations, whole-body fluorescence imaging was performed following oral administration of DiD-labeled Lipo and AS-Lipo in both normal and DSS-induced colitis mice. In vivo fluorescence imaging at 2, 8, and 24 h post-administration revealed distinct biodistribution profiles between the Lipo and AS-Lipo groups (Fig. 5A). In normal mice, fluorescence signals from both formulations showed normal GI transit and clearance, gradually decreasing over 24 h. In contrast, DSS-treated mice exhibited markedly higher and prolonged fluorescence intensity, with AS-Lipo showing the strongest retention in the GI region. This selective accumulation suggests that the inflamed colon microenvironment, characterized by ATX overexpression and increased vascular permeability, facilitates targeting of AS-Lipo. Ex vivo fluorescence imaging of excised GI tissues at 24 h further confirmed enhanced colonic localization of AS-Lipo compared with Lipo (Fig. 5B and Fig. S12). Quantitative fluorescence analysis (Fig. 5C) showed sustained GI signal intensity for AS-Lipo up to 24 h, whereas fluorescence from Lipo rapidly declined. Consistently, colonic fluorescence quantification at 24 h (Fig. 5D) revealed markedly greater accumulation of AS-Lipo in the inflamed colon compared with both Lipo-treated and healthy groups. Moreover, fluorescence imaging of major organs indicated minimal systemic accumulation, supporting limited off-target exposure (Fig. S13). To elucidate the molecular mechanism underlying increased colonic retention, immunofluorescence analysis was performed on colonic tissue sections. Strong DiD signals were distributed throughout the mucosa of 3% DSS-induced mice administered with AS-Lipo (Fig. 5E). Co-immunostaining confirmed increased ATX and F4/80 expression in the inflamed colon, and DiD-AS-Lipo signals were preferentially localized to ATX- or F4/80-rich regions. Line profile analysis further demonstrated synchronized intensity co-peaks between DiD-AS-Lipo and ATX signals, supporting spatial overlap within inflamed regions (Fig. S14A). Similarly, DiD-AS-Lipo showed overlapping intensity distributions with F4/80 and coordinated fluorescence peaks consistent with macrophage-associated accumulation (Fig. S14B). Collectively, these results provide evidence that AS-Lipo preferentially accumulates and persists within ATX- and macrophage-rich inflammatory regions, supporting an ATX-mediated anchoring mechanism for long-term colonic retention.

### The therapeutic effects of AS-Lipo@R on DSS-induced colitis

**Fig. 6.**
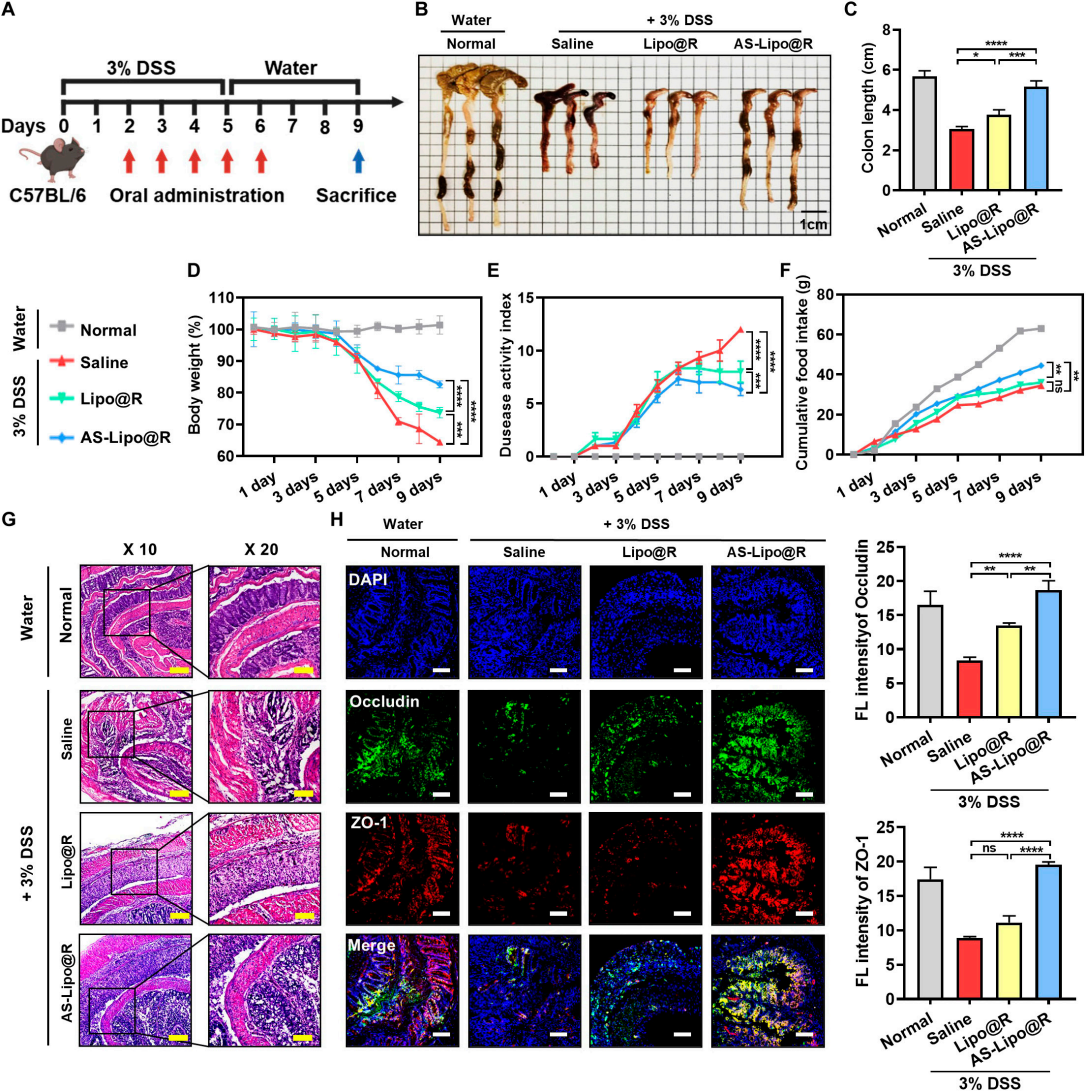
Therapeutic effects of AS-Lipo@R in DSS-induced colitis models. Lipo@R and AS-Lipo@R were orally administered once daily from day 2 to day 6 (lipid: 1.8 μmol, rapamycin: 50 μg), and therapeutic outcomes were evaluated on day 9 (*n* = 3). (A) Schematic of the DSS-induced colitis experimental timeline. (B) Representative macroscopic images of excised colons from each group. (C) Quantification of colon length. (D to F) Daily monitoring of body weight, disease activity index (DAI), and cumulative food intake throughout the treatment period. (G) Representative H&E-stained colon sections showing mucosal architecture and inflammatory damage (scale bars, 100 μm). (H) Immunofluorescence staining of colon sections for tight junction proteins Occludin (green) and ZO-1 (red), with DAPI as a nuclear counterstain (scale bar, 100 μm). Quantification of fluorescence intensity is shown on the right (quantified using ImageJ). Statistical analysis was performed using 1-way or 2-way ANOVA with Tukey’s post hoc test. *P < 0.05, **P < 0.01, ***P < 0.001, and ****P < 0.0001. ns, not significant.

We evaluated the therapeutic efficacy of AS-Lipo@R in an acute DSS-induced colitis model. Colitis was induced by administration of 3% DSS in drinking water for 5 d (days 0 to 5), followed by normal water for 4 d (days 6 to 9). Mice were randomly divided into 4 groups: Normal (water only), 3% DSS + Saline, 3% DSS + Lipo@R, and 3% DSS + AS-Lipo@R (Fig. 6A). Treatments were administered orally once daily from day 2 to day 6 (5 doses), corresponding to the early inflammatory phase during DSS exposure and the immediate recovery period. Disease severity was assessed by multiple clinical indices, including colon length, body weight, DAI, and cumulative food intake. Among these indicators, colon length is widely recognized as a sensitive and direct measure of colonic tissue injury; therefore, we assessed both gross colon morphology and quantitative changes in overall colon length (Fig. 6B and C). As expected, DSS administration resulted in marked colon shortening in the saline-treated group compared with normal controls. Lipo@R treatment produced partial recovery of colon length, whereas AS-Lipo@R restored colon length to near-normal levels, indicating stronger protection against DSS-induced colonic tissue injury. Consistent with these findings, daily monitoring revealed that DSS-treated saline mice exhibited progressive body weight loss, elevated DAI scores, and reduced food intake (Fig. 6D to F). AS-Lipo@R significantly attenuated disease progression, as reflected by improved body weight maintenance, suppressed DAI escalation, and recovery of food intake, compared with saline and Lipo@R-treated groups. To further evaluate tissue-level protection, colon sections were subjected to hematoxylin and eosin (H&E) staining to assess epithelial integrity, crypt structure, inflammatory infiltration, and mucosal architecture (Fig. 6G). Normal mice showed intact crypt organization and preserved mucosal structure with minimal immune cell infiltration. In contrast, DSS + saline mice exhibited severe histopathological features, including crypt loss, epithelial erosion, lamina propria inflammatory infiltration, and pronounced submucosal edema. Lipo@R treatment improved histological features; however, residual mucosal disruption and inflammatory changes remained detectable. Notably, AS-Lipo@R treatment markedly alleviated DSS-induced tissue damage, showing restored crypt architecture, improved epithelial continuity, and reduced inflammatory infiltration. Because epithelial barrier disruption is a key pathological hallmark of colitis, we further evaluated tight junction integrity by immunofluorescence staining of ZO-1 and Occludin (Fig. 6H). Compared with normal controls, DSS + saline mice exhibited substantially reduced ZO-1 and Occludin staining, indicating disruption of tight junction and compromised barrier integrity. In contrast, AS-Lipo@R markedly restored both the fluorescence intensity and junctional localization of these proteins, suggesting recovery of epithelial tight junction structure.

To further clarify the individual contributions of rapamycin and AS-Lipo to these therapeutic outcomes, additional DSS-induced colitis experiments were conducted, including Free R and AS-Lipo control groups (Fig. S15). While both Free R and AS-Lipo alone demonstrated measurable protective effects, AS-Lipo@R consistently produced the most pronounced improvements across clinical parameters, supporting an enhanced therapeutic benefit beyond either component alone. Furthermore, to assess therapeutic relevance under a delayed-intervention (post-onset) setting, AS-Lipo@R was administered after DSS exposure was completed (Fig. S16). Notably, AS-Lipo@R retained significant therapeutic activity even when treatment was initiated after disease establishment, without inducing detectable systemic toxicity as confirmed by serum biochemical analysis under the therapeutic regimen. Histological examination of major organs further revealed no treatment-related abnormalities, as evidenced by normal tissue architecture in H&E-stained sections (Fig. S17). Collectively, these results demonstrate that AS-Lipo@R exerts potent therapeutic effects in DSS-induced acute colitis by improving clinical outcomes, attenuating histopathological damage, and restoring epithelial barrier-associated tight junction proteins. These findings support that membrane-anchored incorporation of BMP-22 enhances the in vivo efficacy of rapamycin-loaded liposomes, likely through inflammation-associated colonic retention consistent with the proposed ATX-targeted delivery strategy.

### ATX suppression-mediated immune restoration in DSS-induced colitis by AS-Lipo@R

**Fig. 7.**
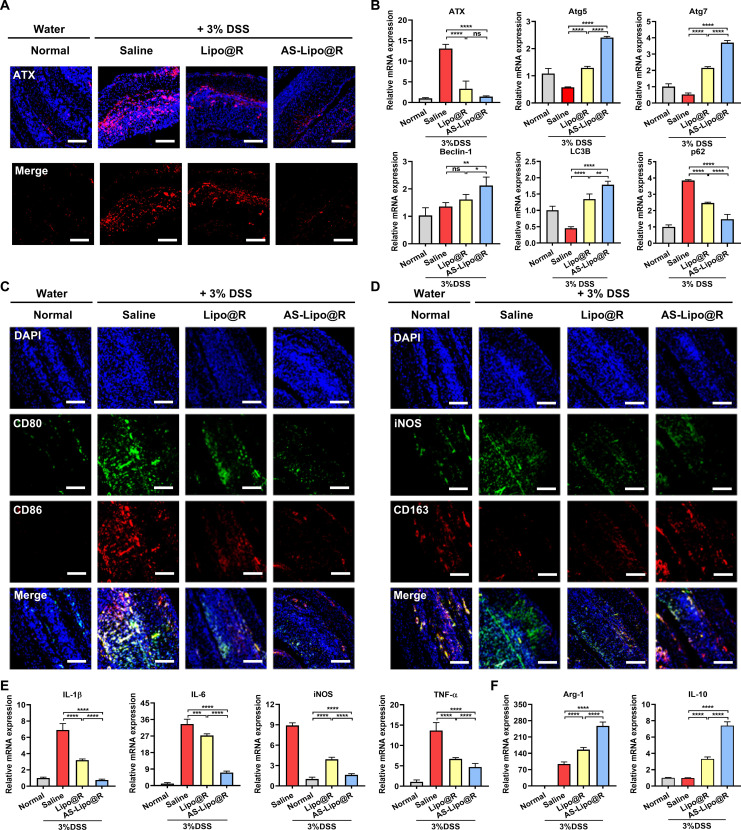
AS-Lipo@R modulates the immune environment in the colon of 3% DSS-induced colitis mice. (A) Immunofluorescence staining of colonic sections showing ATX expression (red) in normal and DSS-treated mice administered with saline, Lipo@R, or AS-Lipo@R. Nuclei were counterstained with DAPI (blue) (scale bar, 50 μm). (B) Relative mRNA expression of ATX and autophagy-related genes (Atg5, Atg7, Beclin-1, LC3B, and p62) in colonic lymph node (CLN) tissues measured by qRT-PCR (*n* = 3). (C) Immunofluorescence staining of colonic sections visualizing M1 macrophage markers CD80 (green) and CD86 (red) in different groups (scale bar, 50 μm). (D) Immunofluorescence staining of M1 (iNOS^+^, green) and M2 (CD163^+^, red) macrophage markers in colonic sections (scale bar, 50 μm). (E and F) Relative mRNA expression of pro-inflammatory cytokines (IL-1β, IL-6, iNOS, TNF-α) and anti-inflammatory cytokines (Arg-1, IL-10) in CLN tissues (*n* = 3). Statistical analysis was performed using one-way ANOVA followed by Tukey’s post hoc test. *P < 0.05, **P < 0.01, ***P < 0.001, and ****P < 0.0001. ns, not significant.

To evaluate whether AS-Lipo@R contributes to immune restoration by modulating ATX signaling in DSS-induced colitis, we first assessed ATX expression by immunofluorescence staining of excised colon tissues (Fig. 7A and Fig. S18). ATX expression was markedly up-regulated in DSS-treated saline controls, whereas AS-Lipo@R treatment substantially reduced ATX immunoreactivity in inflamed colonic tissue, restoring levels toward those observed in normal mice. Next, colonic lymph nodes (CLNs) were assessed. CLNs represent an immunologically active compartment involved in antigen presentation and leukocyte activation, and therefore provide a relevant readout of immune dysregulation in IBD [36]. DSS administration markedly increased ATX expression, indicating pathological activation of the ATX axis under inflammatory conditions (Fig. 7B). Lipo@R induced only a modest reduction, whereas AS-Lipo@R more effectively suppressed ATX expression, supporting that incorporation of a lipid-mimetic ATX inhibitor can attenuate inflammation-associated ATX up-regulation in vivo. Given that dysregulated ATX–LPA signaling has been linked to autophagy suppression, we also examined whether ATX modulation by AS-Lipo@R was associated with recovery of autophagy-related pathways. Analysis of autophagy markers revealed that DSS significantly decreased the level of Atg5, Atg7, Beclin-1, and LC3B while increasing p62, indicating impaired autophagic processing. Lipo@R partially reversed these changes, and AS-Lipo@R decreased p62 expression levels and increased the expression of Atg5, Atg7, Beclin-1, and LC3B, suggesting that autophagy activity was effectively restored under an inflammatory environment. We further investigated whether this immunometabolic remodeling translated into macrophage repolarization in the colonic microenvironment. Immunofluorescence staining for M1 markers CD80 and CD86 (Fig. 7C and Fig. S19) and costaining for iNOS and CD163 (Fig. 7D and Fig. S20) demonstrated that DSS-induced colitis was characterized by increased M1-like polarization (elevated iNOS/CD80/CD86) accompanied by loss of CD163 signal. AS-Lipo@R treatment reversed this inflammatory phenotype, decreasing iNOS while restoring CD163 expression, indicating a shift toward an M2-like anti-inflammatory state. Consistent with these tissue-level findings, qRT-PCR analysis of cytokines in CLN showed that DSS markedly increased transcripts of TNF-α, IL-1β, IL-6, and iNOS, whereas AS-Lipo@R reduced these pro-inflammatory mediators by ~60% to 70% relative to saline controls (Fig. 7E). In parallel, Arg-1 and IL-10 expression was significantly increased in the AS-Lipo@R group, supporting recovery of immunoregulatory signaling (Fig. 7F). Collectively, these results demonstrate that AS-Lipo@R suppresses ATX up-regulation, restores autophagy-related pathways, and promotes macrophage reprogramming toward a pro-resolution immune state in DSS-induced colitis.

## Discussion

In this study, we demonstrated that AS-Lipo@R, an orally administered dual-functional nanotherapeutic co-delivering a membrane-anchored ATX inhibitor and rapamycin, exerts potent therapeutic effects in a DSS-induced colitis model. The combined formulation produced a pronounced synergistic benefit over single-component controls, as evidenced by coordinated improvements in clinical indices, histological architecture, and epithelial barrier integrity. These findings support the therapeutic value of concurrently targeting extracellular ATX–LPA-driven inflammatory signaling and intracellular mTOR/autophagy dysregulation in IBD. Mechanistically, the ATX inhibition behavior of AS-Lipo is best understood as a presentation-dependent effect, where bilayer incorporation promotes local interfacial enrichment of the inhibitor at the lipid water boundary, increasing sustained ATX–inhibitor encounters under subsaturating conditions [37,38]. Collectively, this membrane-anchored ATX targeting strategy may serve as a broadly applicable design principle to improve colonic retention and therapeutic exposure of orally administered liposomal systems [39,40].

The synergistic efficacy of AS-Lipo@R can be explained through convergent regulation along the ATX–LPA–AKT/mTOR–autophagy axis [41]. ATX is the principal extracellular source of LPA, and elevated ATX activity in inflammatory settings leads to sustained LPA–LPAR signaling, which activates AKT and downstream mTOR, a central negative regulator of autophagy [42,43]. Accordingly, excessive ATX–LPA signaling is expected to impose persistent suppression of autophagic flux in inflamed intestinal and immune cells. By inhibiting ATX, AS-Lipo@R reduces local LPA availability and thereby attenuates LPA-driven AKT–mTOR activation, while the co-delivered rapamycin further suppresses mTOR signaling [44]. These convergent effects at the level of mTOR provide a mechanistic basis for the coordinated restoration of autophagy observed in our study, as reflected by increased expression of autophagy-related genes, enhanced LC3 levels, and reduced p62 accumulation. Consistent with this mechanistic framework, AS-Lipo@R induced stronger suppression of phosphorylated AKT and mTOR compared with Lipo@R alone, accompanied by a more pronounced recovery of autophagy-related markers. Importantly, immunocytochemistry (ICC) analyses further confirmed increased LC3 puncta formation together with reduced p62 accumulation, supporting restoration of functional autophagic flux rather than nonspecific autophagosome accumulation [45]. In parallel, AS-Lipo@R more effectively reduced oxidative stress and promoted macrophage immune reprogramming toward an anti-inflammatory phenotype compared with single-component treatments. Given that oxidative stress is a key driver of inflammatory macrophage polarization, the combined normalization of ROS and autophagic flux likely contributed to immune resolution and tissue repair in vivo. These molecular and cellular changes were consistent with the observed therapeutic outcomes, including restored crypt architecture, reduced immune infiltration, and recovery of tight junction proteins such as ZO-1 and Occludin.

While this study demonstrates clear therapeutic and mechanistic advantages of AS-Lipo@R in experimental colitis, certain limitations warrant further investigation. Notably, the current evaluation was performed using an acute DSS-induced colitis model, which primarily reflects epithelial injury-driven inflammation and does not fully recapitulate the chronic, relapsing–remitting nature of human IBD. While the acute DSS model provides a robust and reproducible inflammatory setting for evaluating short-term therapeutic efficacy during active disease phases, it is limited in its ability to predict long-term disease control, relapse prevention, or immune memory-associated pathology. Verification of sustained efficacy in chronic or relapsing colitis models will be an important focus of future studies. In addition, further investigation of adaptive immune regulation, including T cell subset profiling, will help clarify the full immunological impact of AS-Lipo@R. Taken together, this work demonstrates that oral delivery of AS-Lipo@R enables dual targeting of ATX-driven inflammatory signaling and autophagy dysregulation, leading to a synergistic therapeutic benefit in experimental colitis. Importantly, the membrane-anchored incorporation of a lipid-mimetic ATX inhibitor as an ATX-scavenging component represents a unique and generalizable strategy for enhancing inflammation-selective retention of liposomal therapeutics.

In conclusion, this study presents a dual-action strategy for IBD treatment by simultaneously modulating inflammatory signaling and restoring autophagy. We developed AS-Lipo@R by incorporating a lipid-mimetic ATX inhibitor into the liposomal membrane to enable ATX-targeted retention and encapsulating rapamycin to enhance intracellular delivery. AS-Lipo@R promoted anti-inflammatory macrophage repolarization and inhibited ATX activity in vitro while significantly alleviating inflammation and restoring mucosal integrity in a DSS-induced colitis model. Notably, AS-Lipo@R also remained effective in a delayed-treatment setting, supporting its therapeutic relevance in established colitis. This membrane-anchored ATX targeting strategy may be broadly applicable to other liposomal therapeutics by enhancing colonic retention and local drug exposure. Although further validation in chronic models is needed, AS-Lipo@R represents a promising therapeutic platform for IBD.

## Data Availability

All data needed to evaluate the conclusions in the paper are present in the paper and/or the Supplementary Materials. Additional data related to this paper may be acquired upon request from the authors.
