# Supplementary material for: Autotaxin-Scavenging Nanoliposomes for Prolonged Colon Retention and Autophagy-Mediated Mucosal Immune Restoration in Colitis
Source: Biomater Res. 2026 Mar 19;30:0345. doi: 10.34133/bmr.0345 (PMC13000114; doi:10.34133/bmr.0345)
Supplement: Supplementary 1 — Table S1 Figs. S1 to S20 [file bmr.0345.f1.docx]

*Supporting Information*

**Autotaxin-Scavenging Nanoliposomes for Prolonged Colon Retention and Autophagy-Mediated Mucosal Immune Restoration in Colitis**

So Won Jeon^1,2^, Jun Kwon^1,2^, Hee Gyeong Ko^1,2^, Jong Sang Yoon^1,2^, Hee Su Sohn^3^, Jeong-Kee Yoon^4^, Suk-Ho Bhang^5^, Min-Ho Kang^1,2,6^, and Han Young Kim^1,2,6,*^

1 Department of Biomedical-Chemical Engineering, The Catholic University of Korea, Bucheon 14662, Republic of Korea

2 Department of Biotechnology, The Catholic University of Korea, Bucheon 14662, Republic of Korea

3 Division of Pulmonary and Critical Care Medicine, Department of Medicine, Brigham and Women’s Hospital, Harvard Medical School, Boston, MA 02115, USA.

4 Department of Systems Biotechnology, Chung-Ang University, Anseong 17546, Republic of Korea

5 School of Chemical Engineering, Sungkyunkwan University, Suwon 16419, Republic of Korea

6 Research Institute for Controlled Biomaterials of Regulated Cell Death, The Catholic University of Korea, Bucheon 14662, Republic of Korea

*Address correspondence to: [hy0408@catholic.ac.kr](mailto:hy0408@catholic.ac.kr)

**This file includes:**

Supporting Table S1

Supporting Figures S1 to S20

**Table S1**  **Primer Sequences**

| Gene | Primer | Sequence (5’-3’) |
| --- | --- | --- |
| *TNF-α* | Forward | GAT CGG TCC CCA AAG GGA TG |
|  | Reverse | CCA CCT GGT GGT TTG TGA GTG |
| *IL-6* | Forward | AGC TAC CTG GAG TAC ATG AAG A |
|  | Reverse | GTG ACT CCA GCT TAT CTC TTG GT |
| *IL-1β* | Forward | GCCACC TTT TGA CAG TGA TGA G |
|  | Reverse | GAC AGC CCA GGT CAA AGG TT |
| *iNOS* | Forward | TGG AAC ATT CTG TGC TGT CCC |
|  | Reverse | GCA AAA CAT TTC CTG GGA GCG |
| *IL-10* | Forward | GCT CTT GCA CTA CCA AAG CC |
|  | Reverse | CTG CTG ATC CTC ATG CCA GT |
| *Occludin* | Forward | TGAAAGTCCACCTCCTTACAGA |
|  | Reverse | CCGGATAAAAAGAGTACGCTGG |
| *ZO-1* | Forward | GCTTTAGCGAACAGAAGGAGC |
|  | Reverse | TTCATTTTTCCGAGACTTCACCA |
| *Arg-1* | Forward | GAT TAT CGG AGC GCC TTT CT |
|  | Reverse | CCA CAC TGA CTC TTC CAT TCT T |
| *ATX* | Forward | ACT TTT GCC GTT GGA GTC AAT |
|  | Reverse | GGA GTC TGA TAG CAC TGT AGG |
| *Atg 5* | Forward | GAT GCG GTT GAG GCT CAC |
|  | Reverse | CTG TCA TTC TGC AGT CCC ATC |
| *Atg 7* | Forward | AGC CTG TTC ACC CAA AGT TC |
|  | Reverse | CAT GTC CCA GAT CTC AGC AG |
| *LC3B* | Forward | GAT AAT CAG ACG GCG CTT GC |
|  | Reverse | TCT CAC TCT CGT ACA CTT CGG |
| *Beclin-1* | Forward | AAT CTA AGG AGT TGC CGT TAT AC |
|  | Reverse | CCA GTG TCT TCA ATC TTG CC |
| *p62* | Forward | GCT CAG GAG GAG ACG ATG AC |
|  | Reverse | AGA AAC CCA AGG ACA GCA TC |
| β-actin | Forward | GGC TAT ATT CCC CTC CAT CG |
|  | Reverse | CCA GTT GGT AAC AAT GCC ATG T |


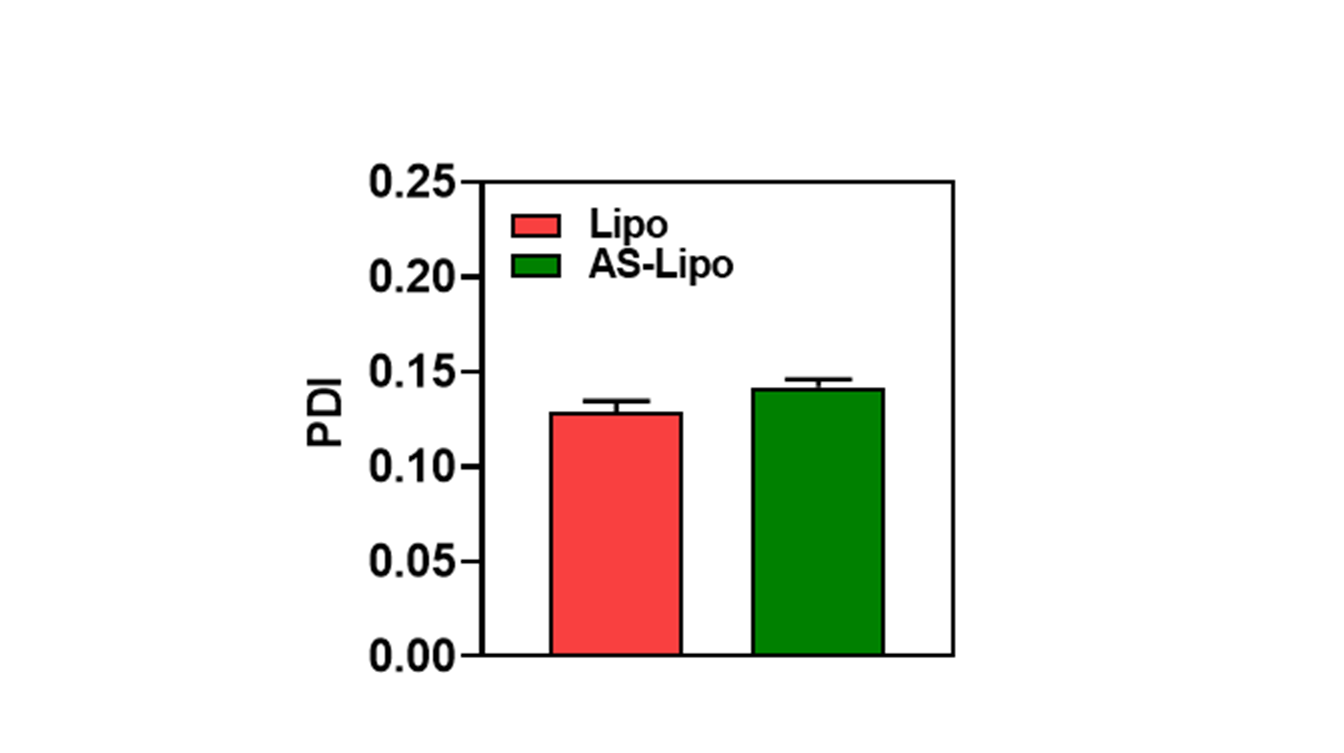


**Fig. S1.** Polydispersity index (PDI) of liposomal formulations. The PDI of Lipo and AS-Lipo was determined by dynamic light scattering (DLS), showing a narrow size distribution and high uniformity of the liposomal populations. Data are presented as mean ± SEM (n = 3).

**
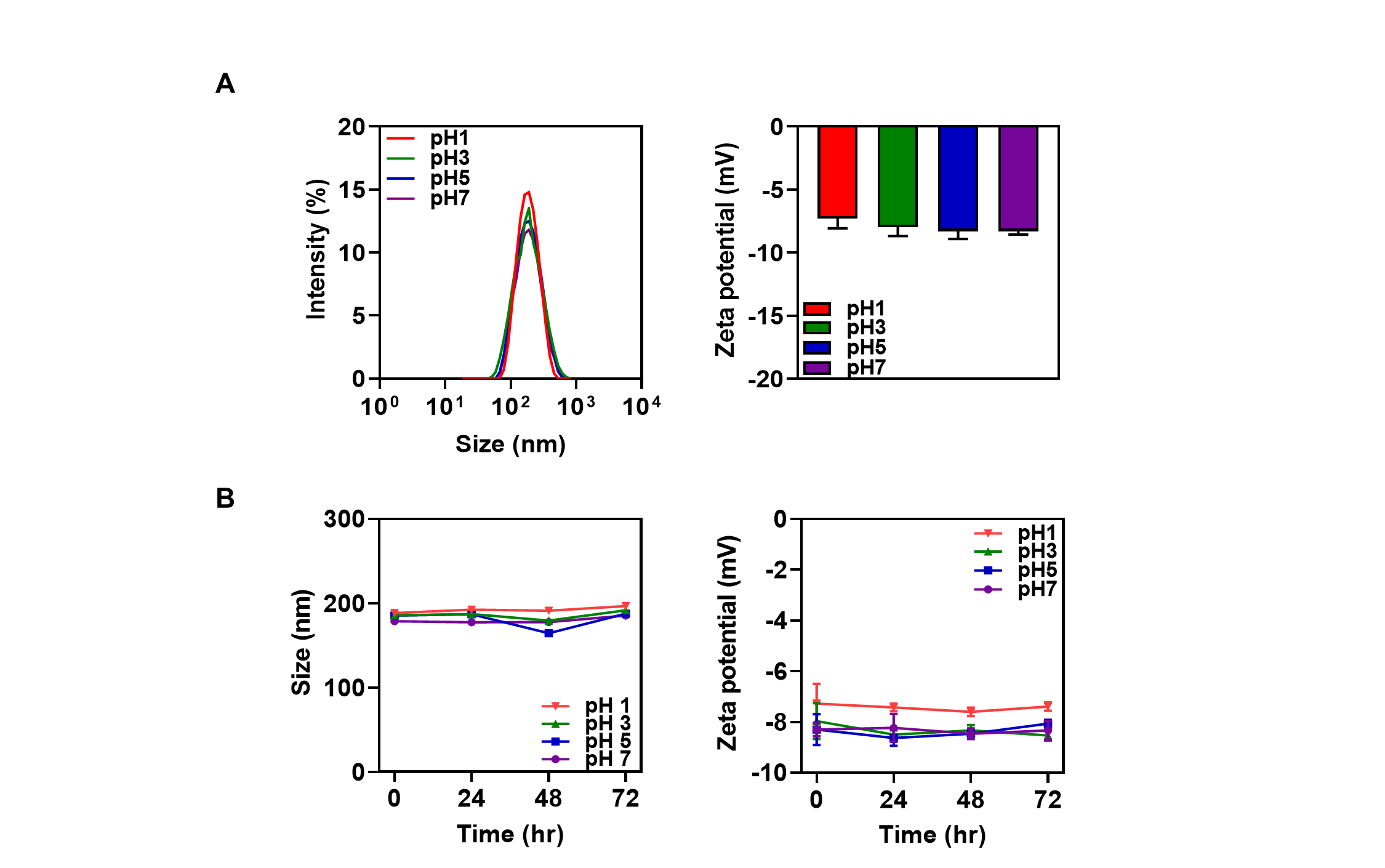
Fig. S2.** pH-dependent physicochemical stability of AS-Lipo@R. (A) Particle size distribution and z-potential of AS-Lipo@R measured at pH 1, 3, 5, and 7 by DLS. (B) Time-dependent stability of AS-Lipo@R under different pH conditions over 72 h, monitored by particle size and z-potential measurements using DLS. Data are presented as mean ± SEM (n = 3).


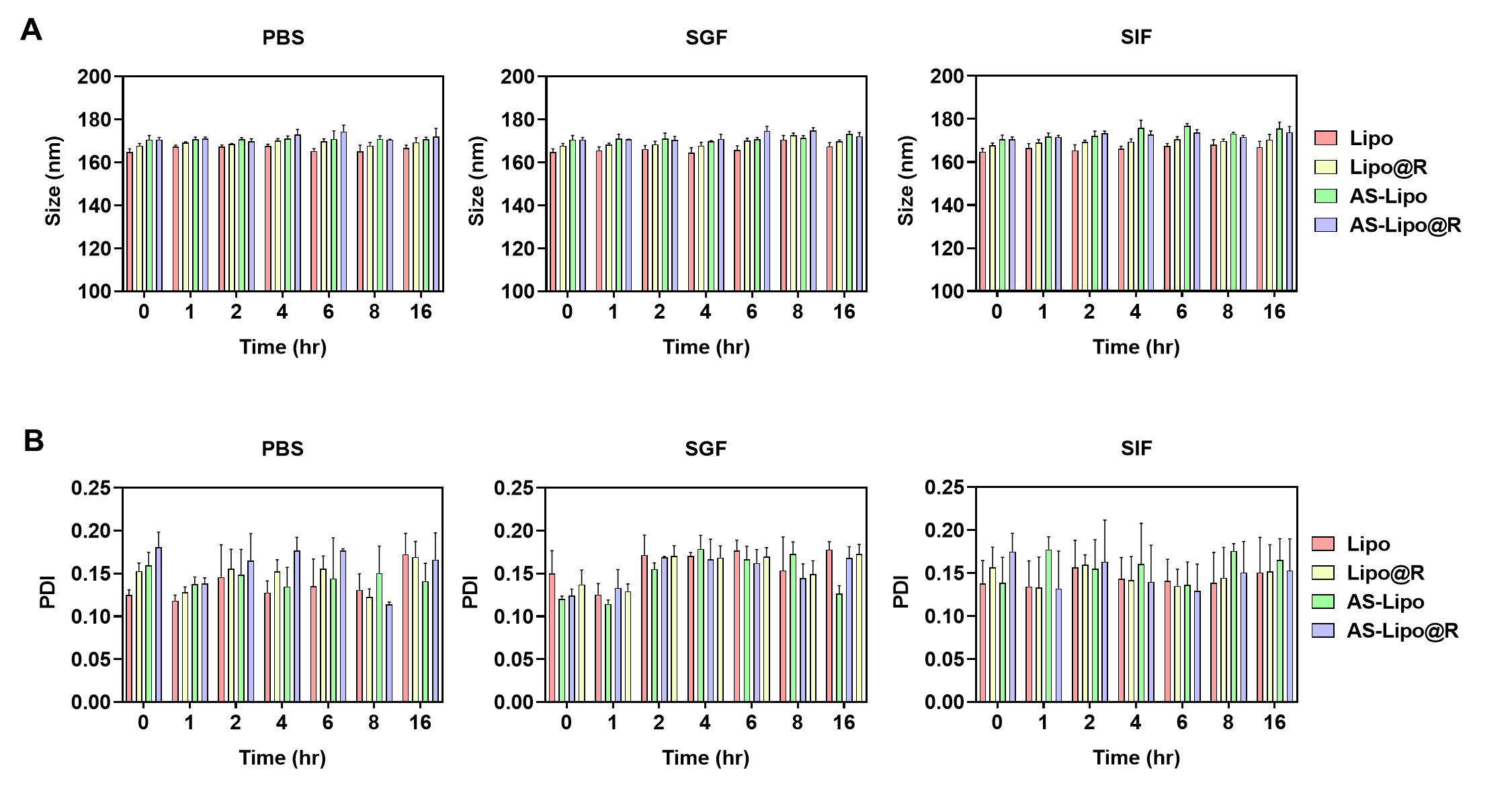


**Fig. S3.** Gastrointestinal stability of liposomal formulations under simulated GI conditions. (A) Time-dependent changes in hydrodynamic particle size of Lipo, Lipo@R, AS-Lipo, and AS-Lipo@R during incubation in PBS, simulated intestinal fluid (SIF), and simulated gastric fluid (SGF) for up to 16 h. (B) Corresponding PDI values of each formulation under the same conditions. All formulations maintained stable particle sizes and narrow PDI distributions without significant aggregation in PBS, SIF, or SGF, indicating preserved structural integrity of the liposomal bilayer during simulated gastrointestinal transit. Data are presented as mean ± SD (n = 3).


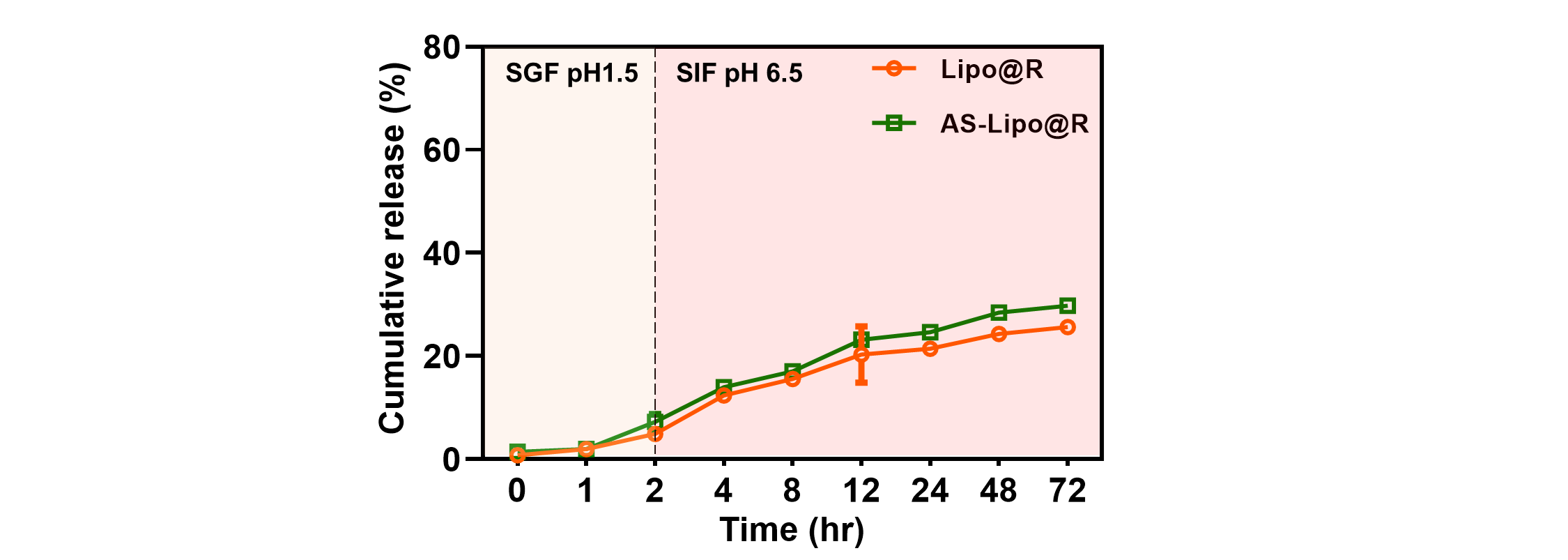


**Fig. S4.** GI-relevant in vitro release profile of rapamycin from liposomal formulations. Cumulative release of rapamycin from Lipo@R and AS-Lipo@R under sequential simulated gastrointestinal conditions. Formulations were first incubated in simulated gastric fluid (SGF, pH 1.5) and subsequently transferred to simulated intestinal fluid (SIF, pH 6.5), as indicated by the dashed vertical line. Rapamycin release was quantified over 72 h, showing minimal premature release in SGF followed by sustained release in SIF. Data are presented as mean ± SD (n = 3).


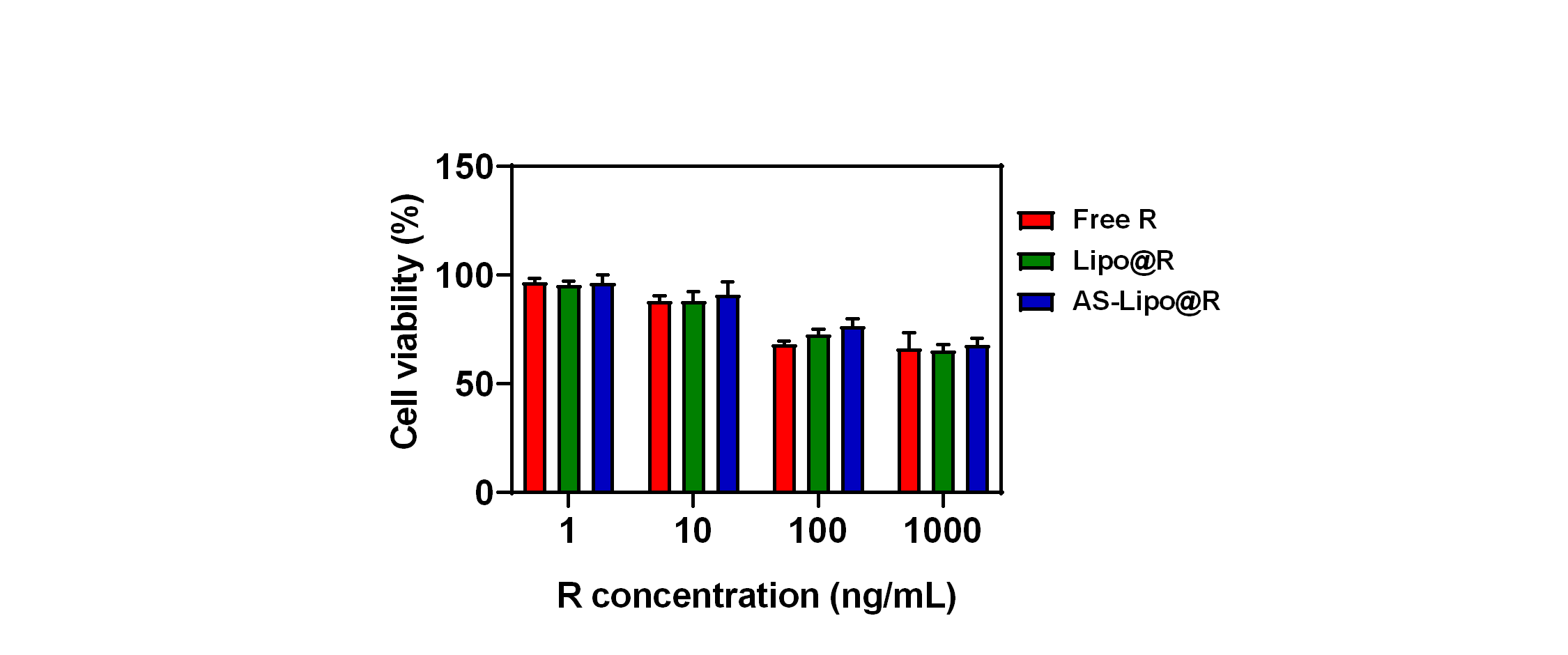


**Fig. S5.** Cytotoxicity evaluation of rapamycin-loaded liposomal formulations. Cell viability of RAW264.7 macrophages after treatment with rapamycin (1–1000 ng/mL) in Free R, Lipo@R, or AS-Lipo@R formulations. Cell viability was assessed by the CCK-8 assay. Data are presented as mean ± SD (n = 3). Statistical significance was determined by one-way ANOVA followed by Tukey’s post hoc test. *p < 0.05, **p < 0.01, ***p < 0.001, ****p < 0.0001.


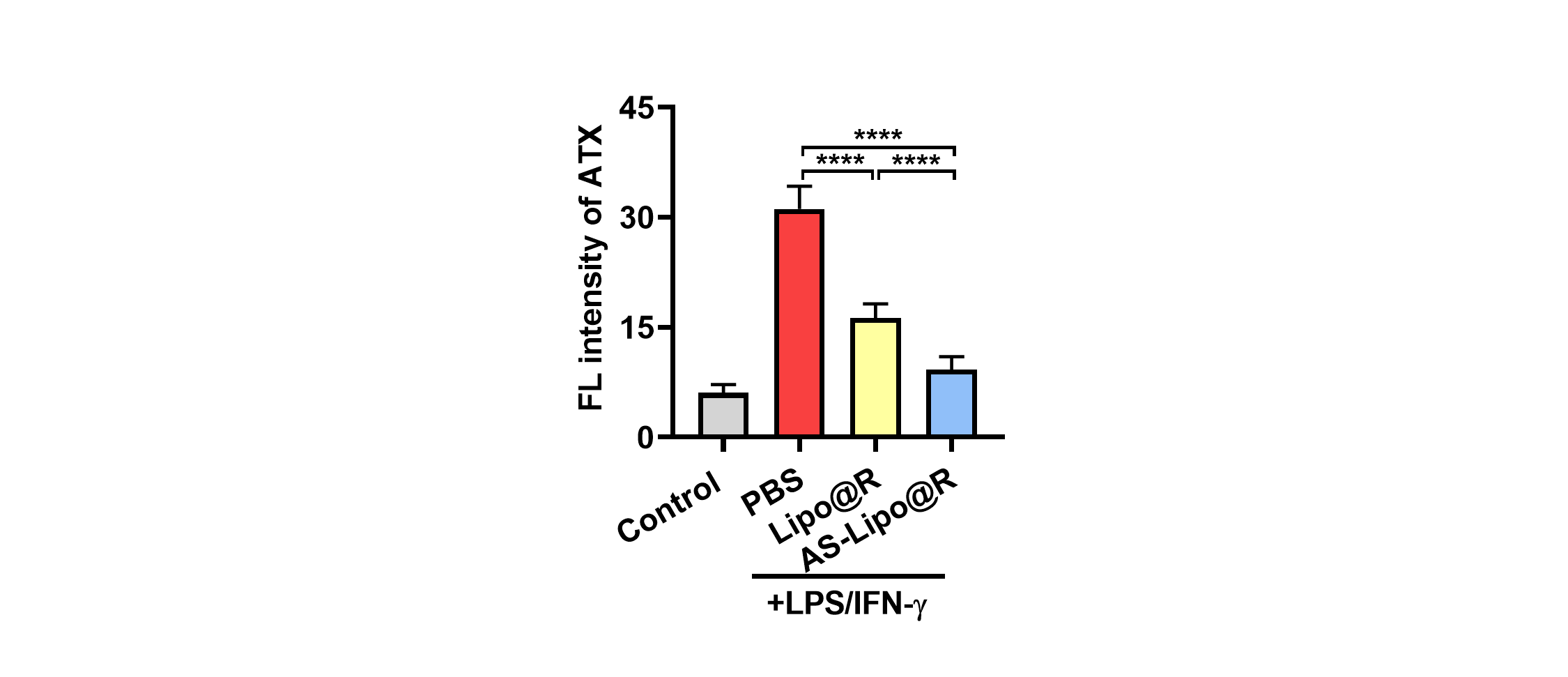


**Fig. S6.** Quantification of ATX fluorescence intensity in LPS/IFN-γ-stimulated RAW264.7 macrophages. Fluorescence intensity of ATX was quantified in RAW264.7 cells treated with PBS, Lipo@R, or AS-Lipo@R under inflammatory conditions induced by LPS/IFN-γ. Data are presented as mean ± SD (n = 3). Statistical significance was determined by one-way ANOVA followed by Tukey’s post hoc test. *p < 0.05, **p < 0.01, ***p < 0.001, ****p < 0.0001


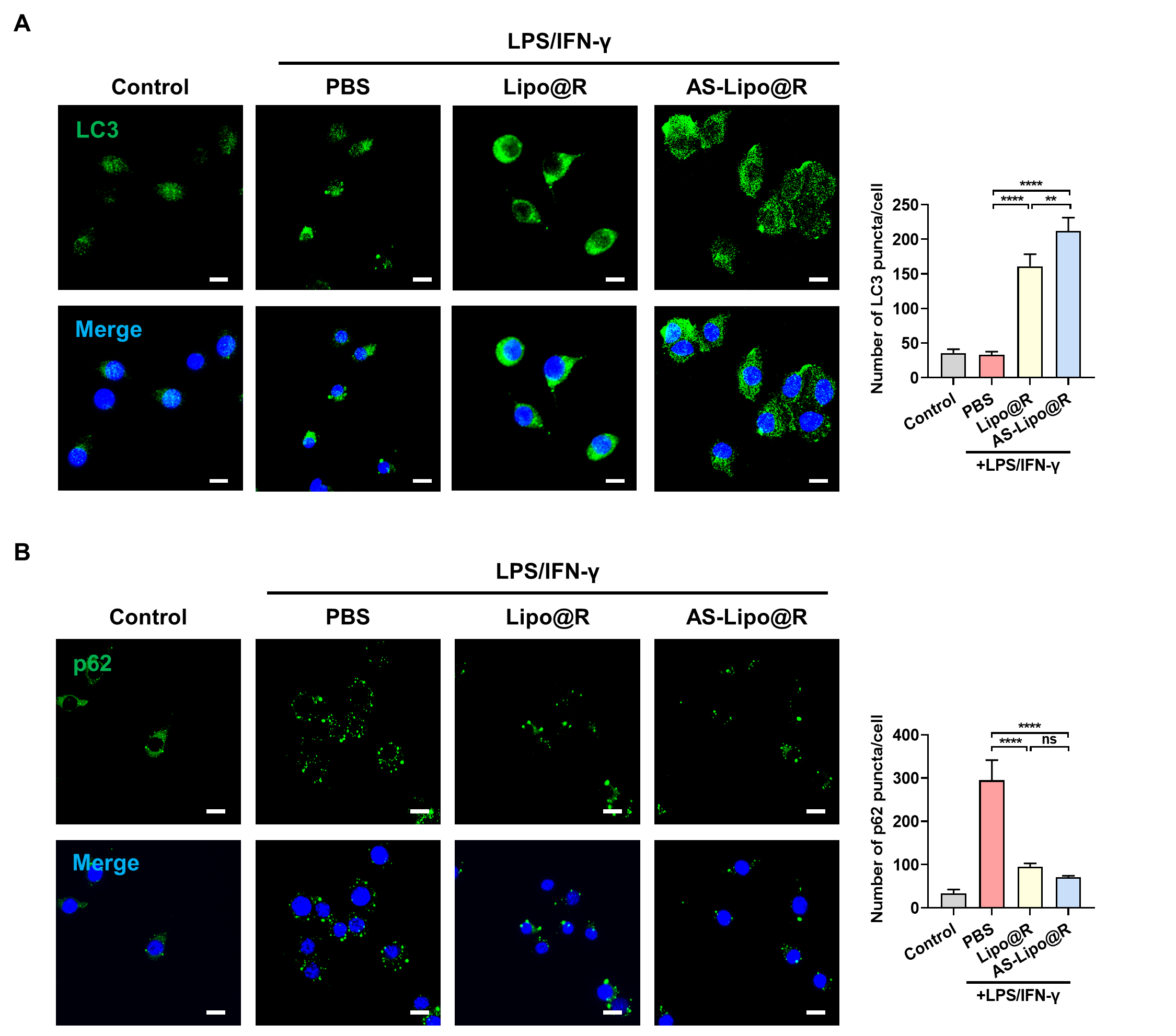


**Fig. S7.** Immunocytochemical evidence of autophagy restoration in inflammatory macrophages. RAW264.7 macrophages were stimulated with LPS/IFN-γ followed by treatment with PBS, Lipo@R, or AS-Lipo@R. Representative ICC images of LC3 puncta formation (A) and p62 puncta accumulation (B) are shown (green), with nuclei counterstained by DAPI (blue). Scale bar, 10 μm. Puncta were quantified on a per-cell basis by normalizing total puncta counts to the number of DAPI-stained nuclei per field (≥3 independent experiments). Data are presented as mean ± SEM. Statistical significance was determined by one-way ANOVA with Tukey’s post hoc test.*p < 0.05, **p < 0.01, ***p < 0.001, ****p < 0.0001; ns, not significant.


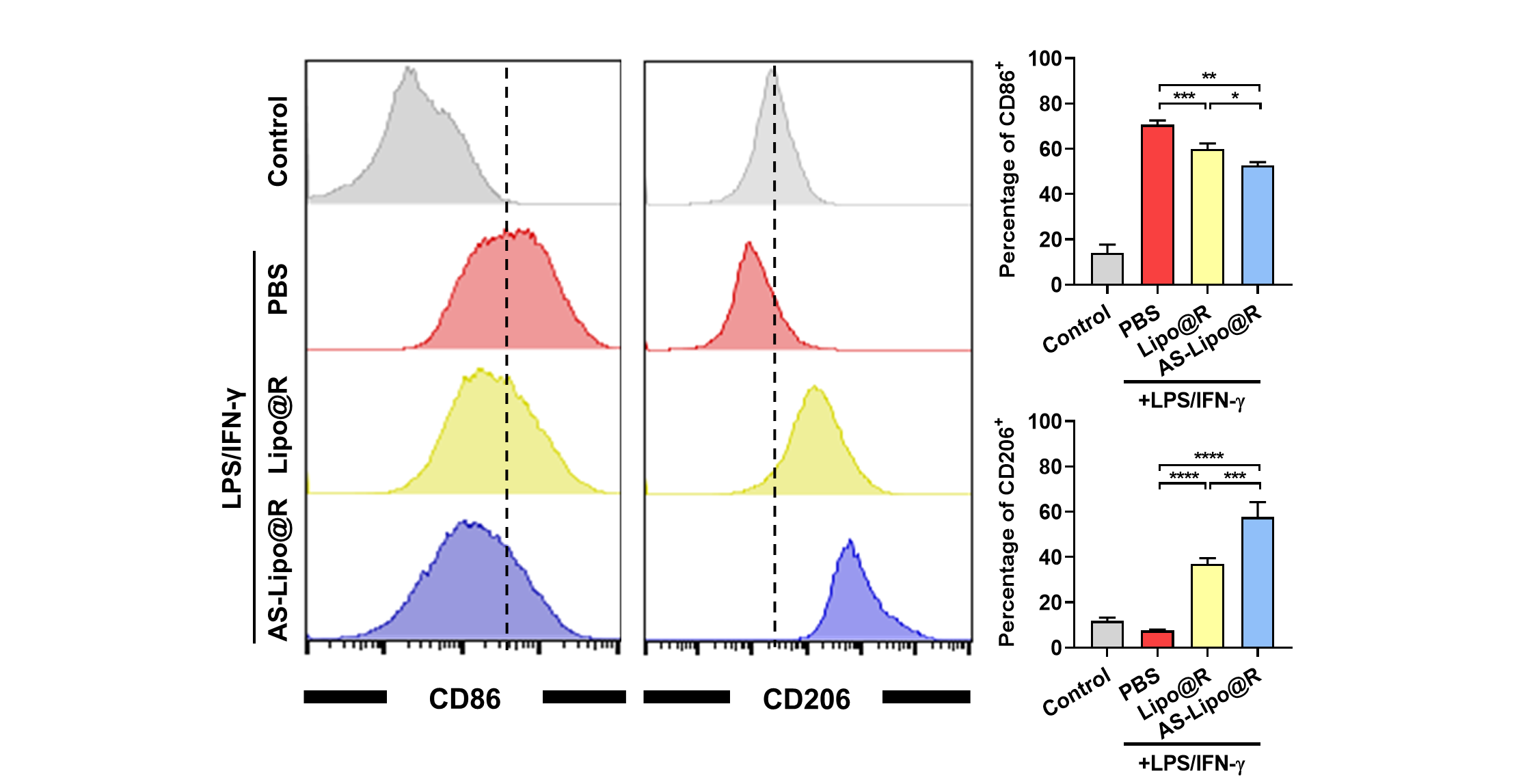


**Fig.S8.** Flow cytometric quantification of macrophage polarization following AS-Lipo@R treatment. Flow cytometry was performed to evaluate macrophage polarization in RAW264.7 cells stimulated with LPS/IFN-γ and treated with PBS, Lipo@R, or AS-Lipo@R. Representative histogram overlays show expression of the M1 marker CD86 (PE) and the M2 marker CD206 (APC). The same gating threshold (dashed line) was applied across all groups. Bar graphs present the percentages of CD86⁺ and CD206⁺ cells (n = 3). Data are presented as mean ± SD. Statistical significance was determined by one-way ANOVA with Tukey’s post hoc test. *p < 0.05, **p < 0.01, ***p < 0.001, ****p < 0.0001; ns, not significant.


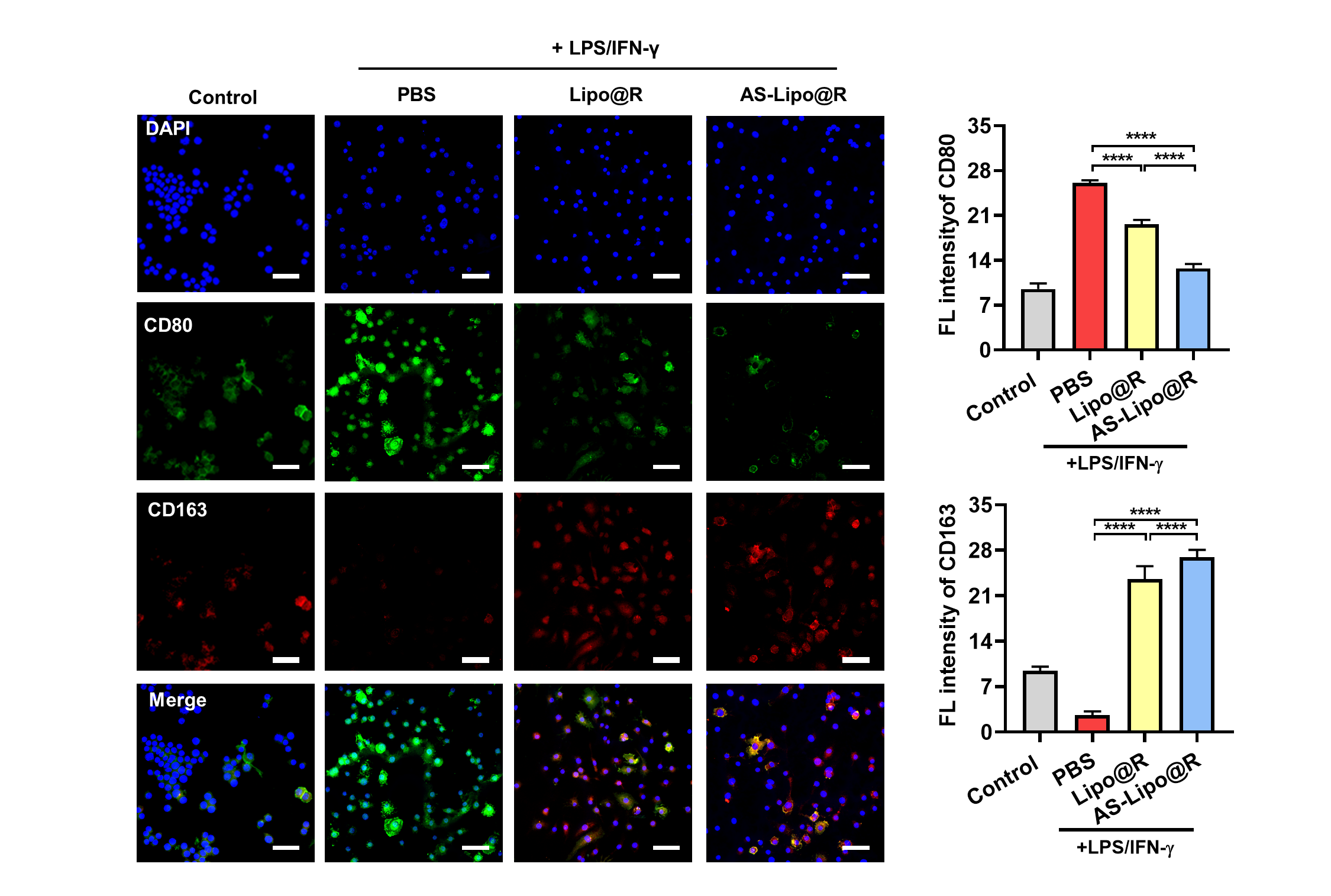


**Fig. S9.** Immunofluorescence analysis of macrophage polarization following AS-Lipo@R treatment. Representative immunofluorescence images of CD80 (M1 marker, green) and CD163 (M2 marker, red) in RAW264.7 macrophages stimulated with LPS/IFN-γ and treated with PBS, Lipo@R, or AS-Lipo@R. Nuclei were counterstained with DAPI (blue). Scale bar: 100 μm. Quantification of fluorescence intensity for CD80 and CD163 is shown on the right (n = 3). Data are presented as mean ± SD. Statistical significance was determined by one-way ANOVA with Tukey’s post hoc test. *p < 0.05, **p < 0.01, ***p < 0.001, ****p < 0.0001.


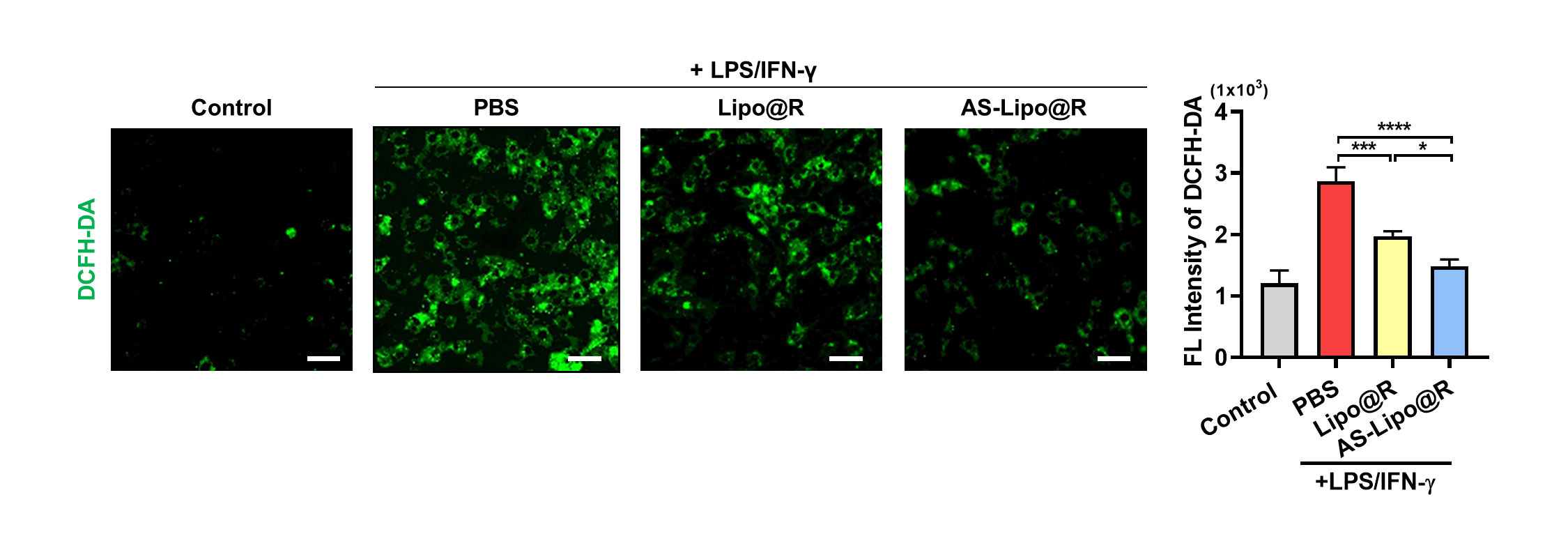


**Fig. S10.** Intracellular ROS levels in activated macrophages following AS-Lipo@R treatment. Representative fluorescence images of DCFH-DA staining in RAW264.7 macrophages stimulated with LPS/IFN-γ and treated with PBS, Lipo@R, or AS-Lipo@R. Quantification of DCF fluorescence intensity is shown on the right (n = 3). Scale bar: 100 μm. Data are presented as mean ± SD. Statistical significance was determined by one-way ANOVA with Tukey’s post hoc test. *p < 0.05, **p < 0.01, ***p < 0.001, ****p < 0.0001.


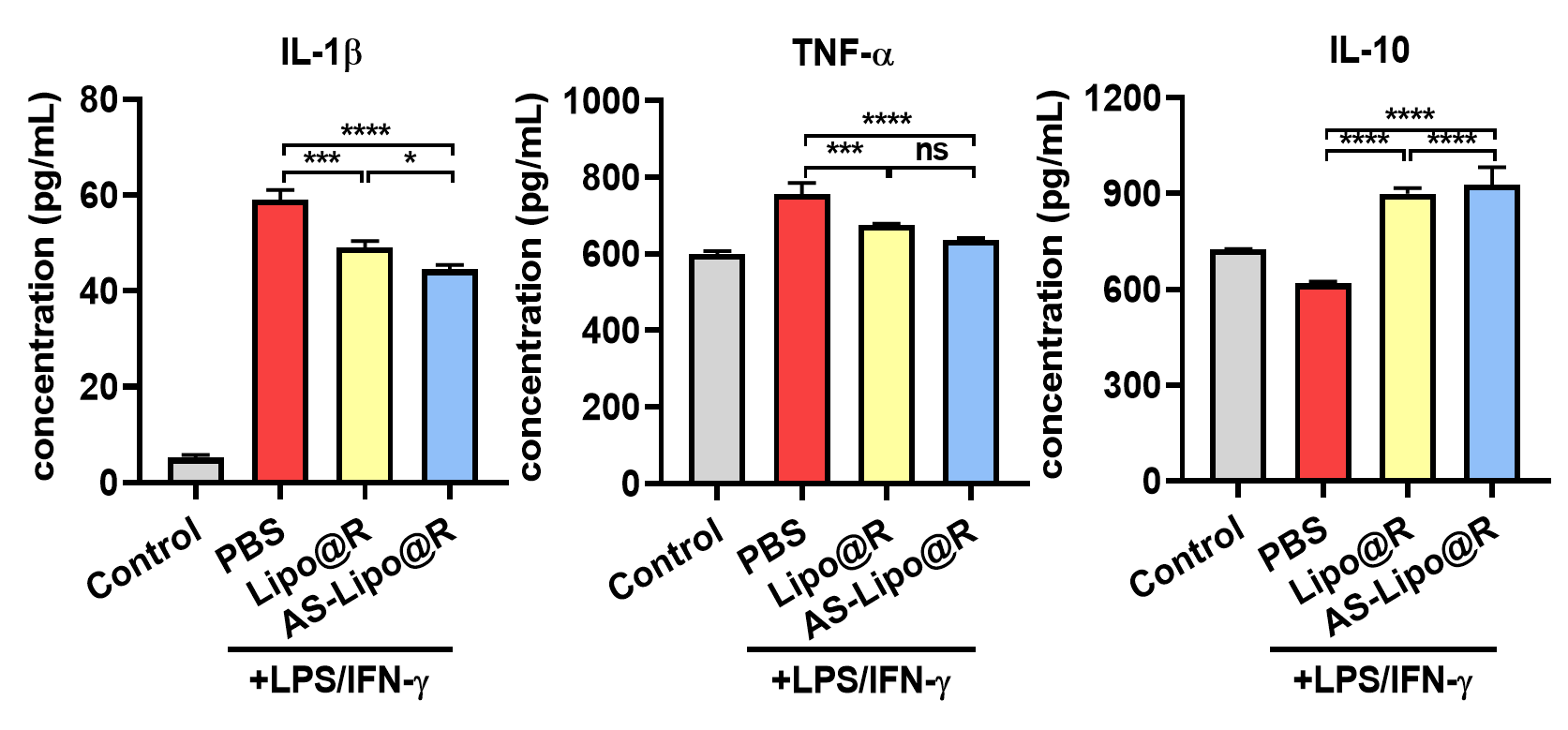


**Fig. S11.** Cytokine secretion profiles of activated macrophages following AS-Lipo@R treatment.

RAW264.7 macrophages were stimulated with LPS/IFN-γ and treated with PBS, Lipo@R, or AS-Lipo@R. The concentrations of pro-inflammatory cytokines (IL-1β and TNF-α) and anti-inflammatory cytokine (IL-10) in the culture supernatants were quantified by ELISA (pg/mL, n = 3). Data are presented as mean ± SD. Statistical significance was determined by one-way ANOVA with Tukey’s post hoc test. *p < 0.05, **p < 0.01, ***p < 0.001, ****p < 0.0001; ns, not significant.

**
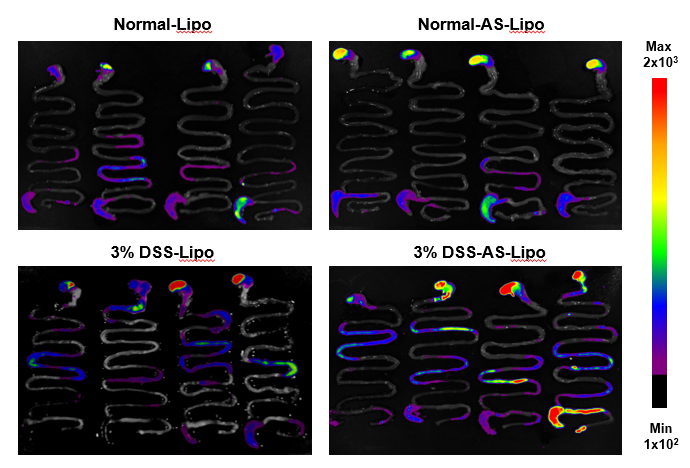
**

**Fig. S12.** Ex vivo biodistribution of DiD-labeled liposomes in normal and DSS-induced colitis mice. Ex vivo fluorescence imaging of the gastrointestinal tract was performed 24 h after oral administration of DiD-labeled Lipo or DiD-AS-Lipo in normal mice and mice with 3% DSS–induced colitis (n = 4 per group). Representative fluorescence images illustrate the intestinal distribution patterns of DiD-Lipo and DiD-AS-Lipo under both healthy and inflammatory conditions. Pseudocolor intensity represents relative fluorescence signal strength, as indicated by the color scale. Enhanced accumulation and prolonged intestinal retention of DiD-AS-Lipo were observed in DSS-treated mice compared with non-targeted DiD-Lipo.


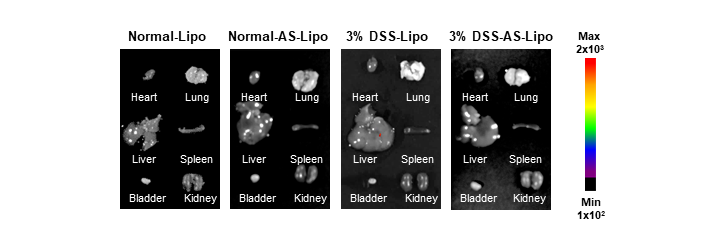


**Fig. S13.** Ex vivo organ biodistribution of DiD-labeled liposomes in normal and DSS-induced colitis mice. Ex vivo fluorescence imaging of major organs (heart, lung, liver, spleen, bladder, and kidney) was performed 24 h after oral administration of DiD-labeled Lipo or DiD-AS-Lipo in normal mice and mice with 3% DSS–induced colitis (n = 4 per group). Representative images display the organ-level biodistribution patterns of DiD-Lipo and DiD-AS-Lipo under both healthy and inflammatory conditions. Pseudocolor intensity indicates relative fluorescence signal strength, as shown in the color scale.


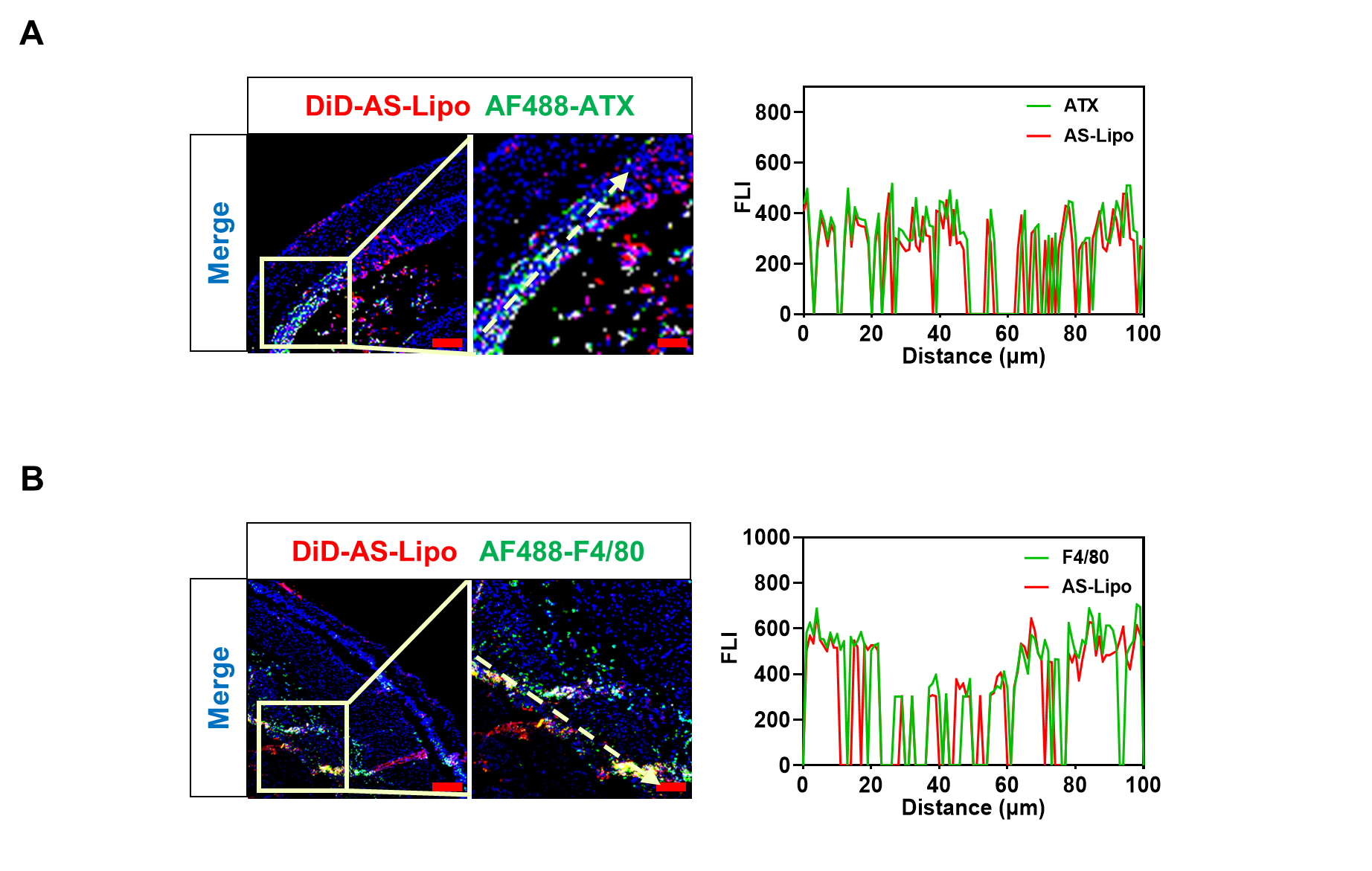


**Fig. S14.** Line profile analysis of colocalization between DiD-AS-Lipo and ATX/F4/80 in 3%DSS-induced colon. (A) Representative merged immunofluorescence images of colonic tissue sections from 3% DSS-induced colitis mice treated with DiD-labeled AS-Lipo, co-stained with ATX (AF488, green; upper panel) or F4/80 (AF488, green; lower panel). Nuclei were counterstained with DAPI (blue). (B)The boxed region in each merged image was magnified, and the fluorescence intensity along the indicated line (white arrow) was plotted as a line profile, showing the spatial overlap between DiD-AS-Lipo (red) and ATX or F4/80 (green) signals. Scale bars are shown in each panel.


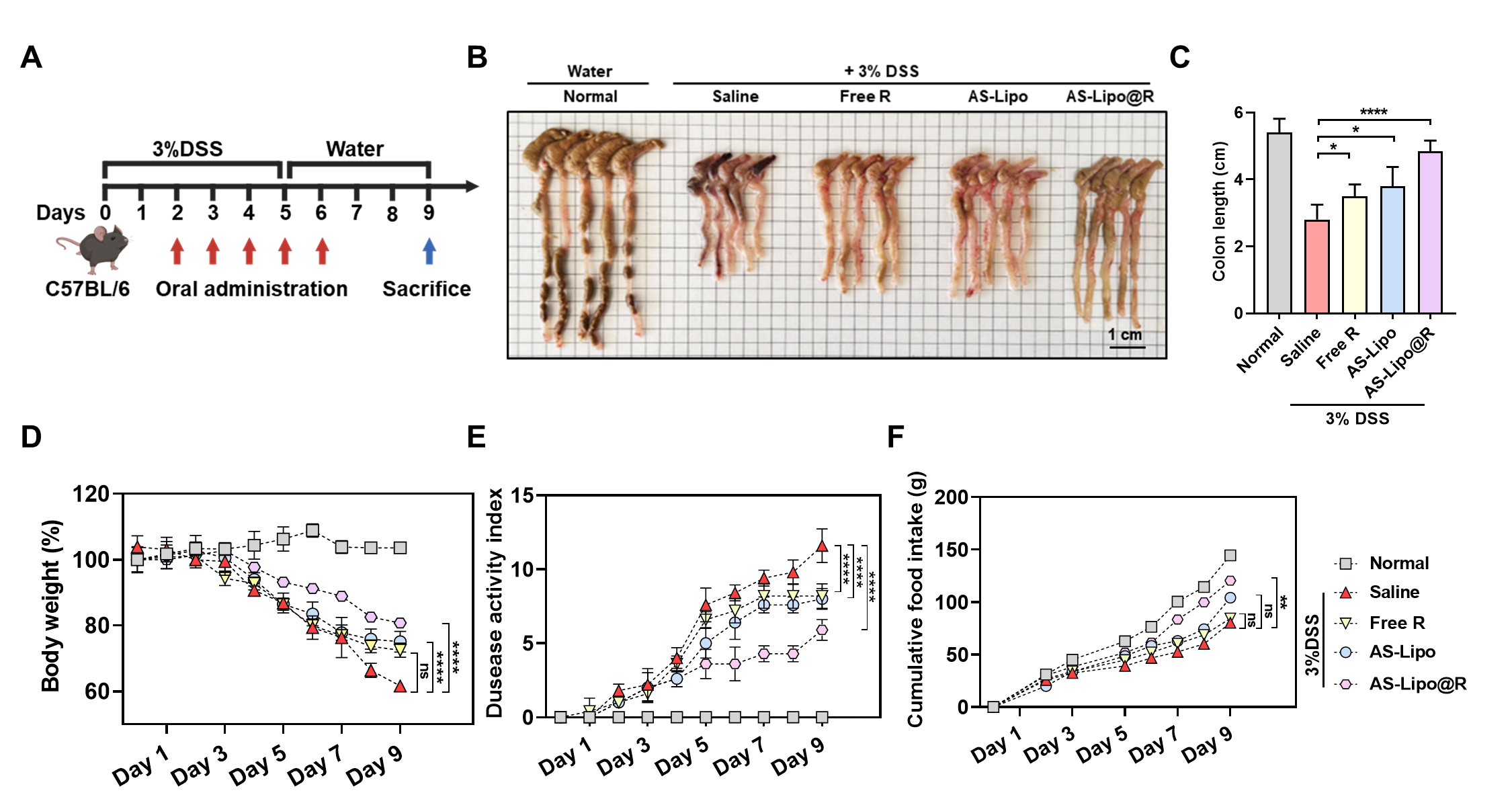


**Fig. S15.** Therapeutic efficacy of AS-Lipo and AS-Lipo@R in a DSS-induced acute colitis model.

(A) Schematic illustration of the experimental design. Acute colitis was induced in C57BL/6 mice by administering 3% DSS in drinking water for 5 consecutive days, followed by a recovery phase with normal water. Saline, Free R, AS-Lipo, or AS-Lipo@R was orally administered once daily during the indicated period. Mice were sacrificed on day 9. (B) Representative images of excised colons from normal mice and DSS-treated mice receiving saline, Free R, AS-Lipo, or AS-Lipo@R. Scale bar: 1 cm. (C) Quantification of colon length at sacrifice. (D) Body weight changes over the experimental period, expressed as a percentage of initial body weight. (E) DAI monitored daily based on body weight loss, stool consistency, and rectal bleeding. (F) Cumulative food intake during the experimental period. Data are presented as mean ± SD (n = 5). Statistical significance was determined using one-way or two-way ANOVA followed by Tukey’s post hoc test. *p < 0.05, **p < 0.01, ***p < 0.001, ****p < 0.0001; ns, not significant.


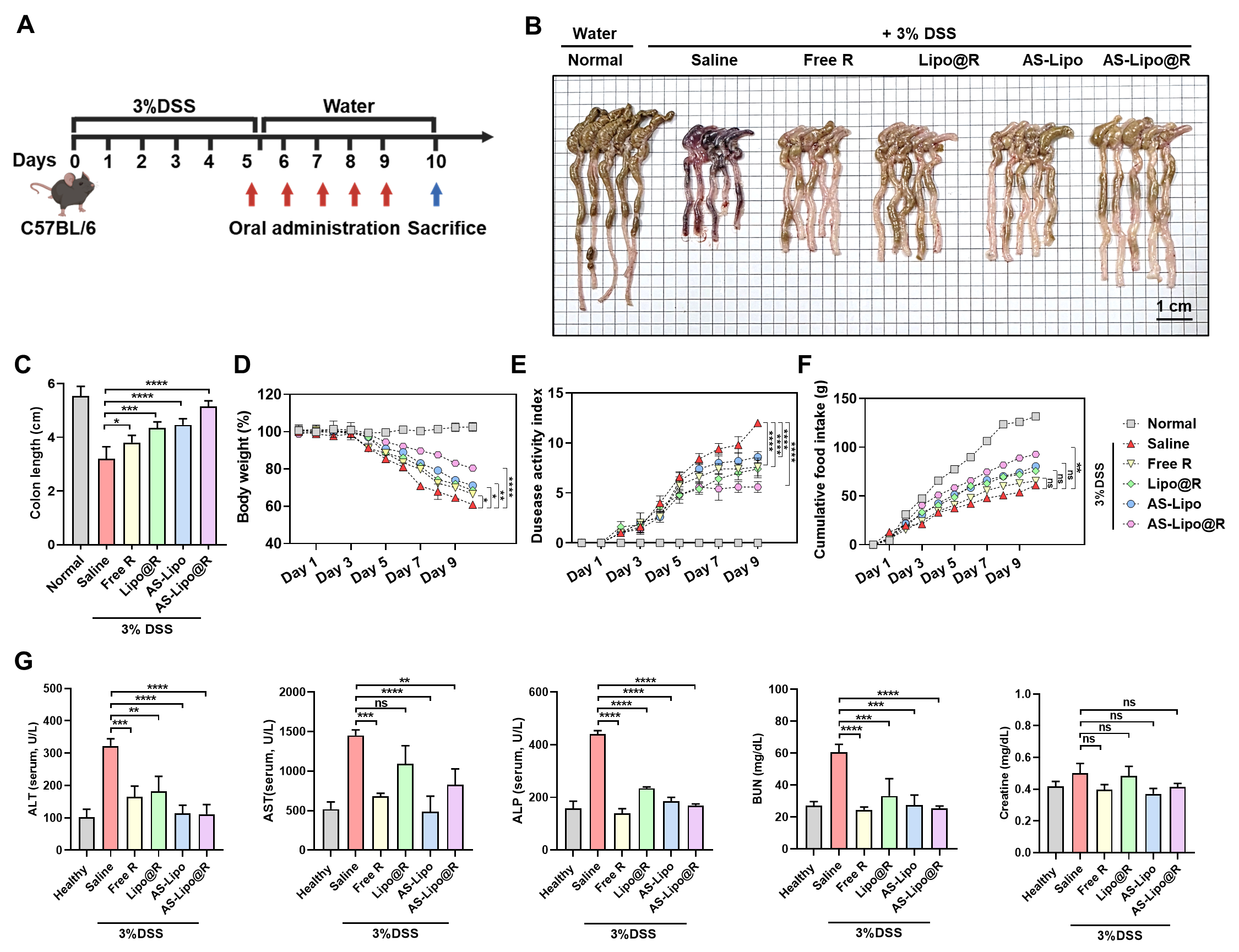


**Fig. S16.** Therapeutic efficacy of AS-Lipo@R under delayed administration in the DSS-induced colitis model. Acute colitis was induced in C57BL/6 mice by administration of 3% DSS in drinking water for 5 consecutive days, followed by replacement with normal water. AS-Lipo@R was orally administered once daily during the recovery phase (days 5–9), and therapeutic outcomes were evaluated on day 10 (n = 5). (A) Schematic illustration of the delayed-treatment experimental timeline. (B) Representative images of excised colons from each group. (C) Quantification of colon length. (D–F) Daily monitoring of body weight change, disease activity index (DAI), and cumulative food intake throughout the experimental period. (G) Serum biochemical parameters (ALT, AST, ALP, BUN, and creatinine) were measured at the endpoint to evaluate systemic toxicity. Data are presented as mean ± SD. Statistical significance was determined using one-way ANOVA (or two-way ANOVA for time-course data) followed by Tukey’s post hoc test. *p < 0.05, **p < 0.01, ***p < 0.001, ****p < 0.0001; ns, not significant.


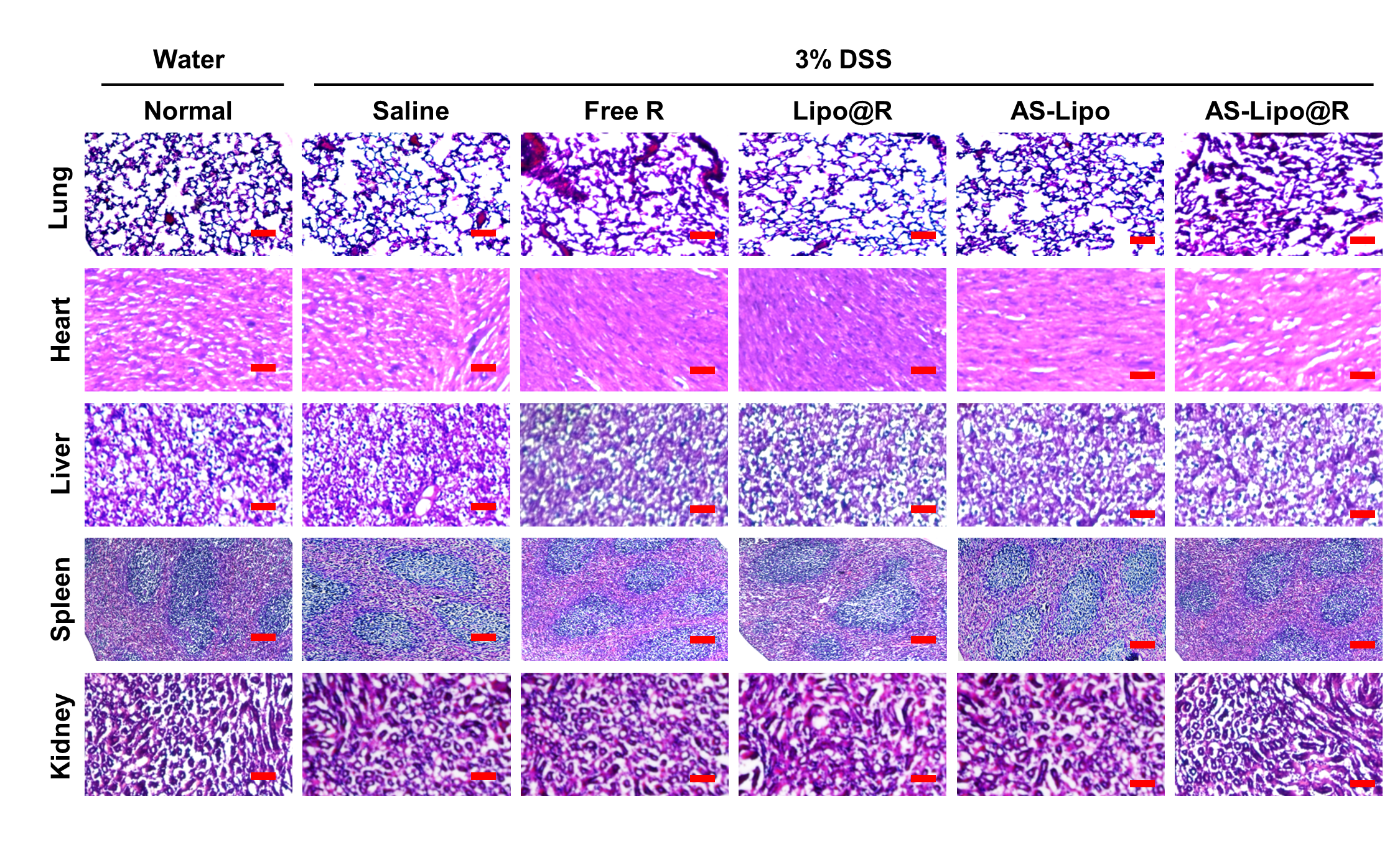


**Fig. S17.** Histological biosafety evaluation of major organs after oral administration in the DSS-induced colitis model. Representative hematoxylin and eosin (H&E)-stained sections of major organs (lung, heart, liver, spleen, and kidney) collected from mice subjected to the DSS-induced colitis experiment, including Normal and 3% DSS-treated groups receiving saline, Free R, Lipo@R, AS-Lipo, or AS-Lipo@R. No evident tissue injury, inflammatory cell infiltration, or pathological abnormalities were observed in any treatment group under the dosing regimen used. Scale bars are shown in each panel.


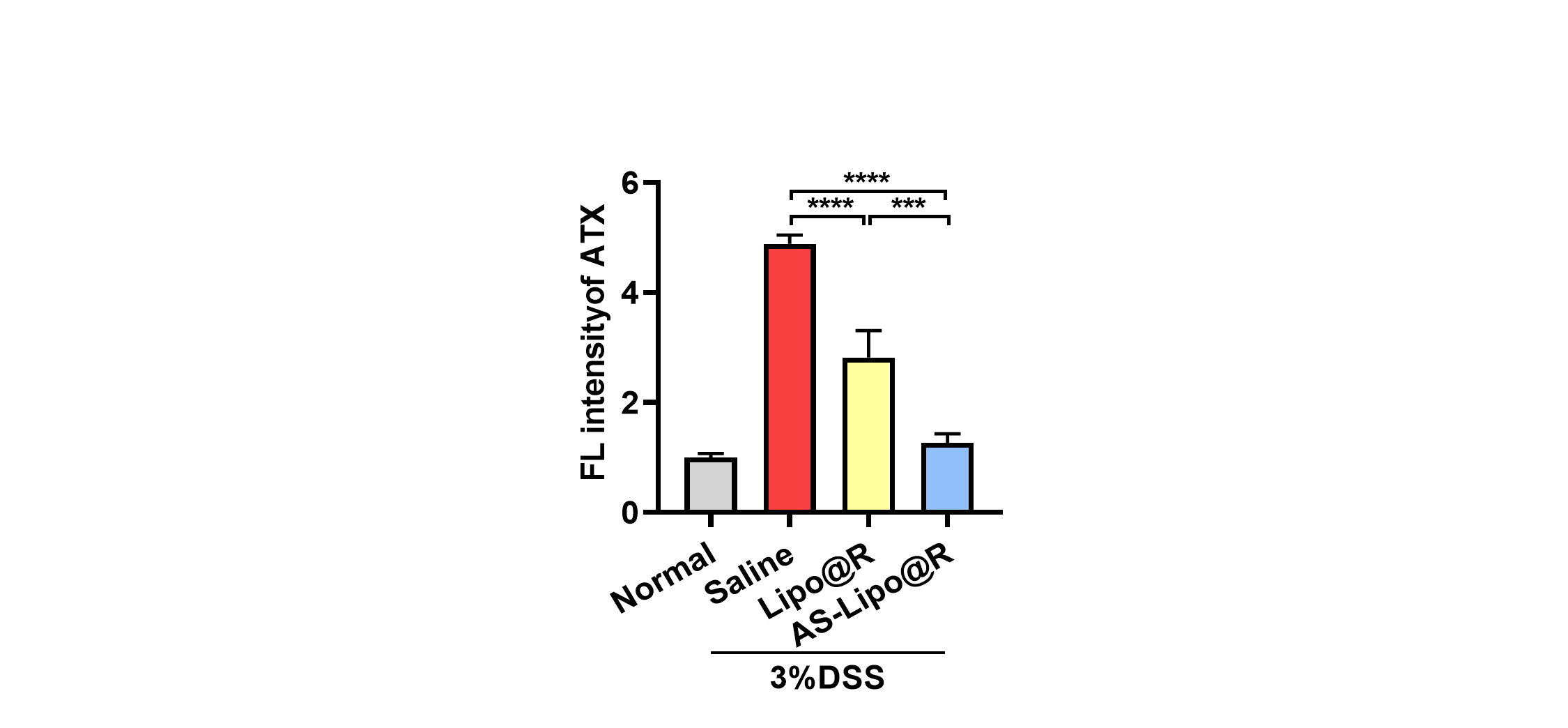
**Fig. S18.** Quantitative analysis of ATX expression in colonic tissues. Representative ATX immunofluorescence staining was quantified in colon sections to evaluate ATX expression in DSS-induced colitis. Fluorescence intensity of ATX was measured from randomly selected fields of colonic mucosa and normalized to the tissue area (n = 3 mice per group). Data are presented as mean ± SD. Statistical significance was determined using one-way ANOVA followed by Tukey’s post hoc test. *p < 0.05, **p < 0.01, ***p < 0.001, ****p < 0.0001.

**
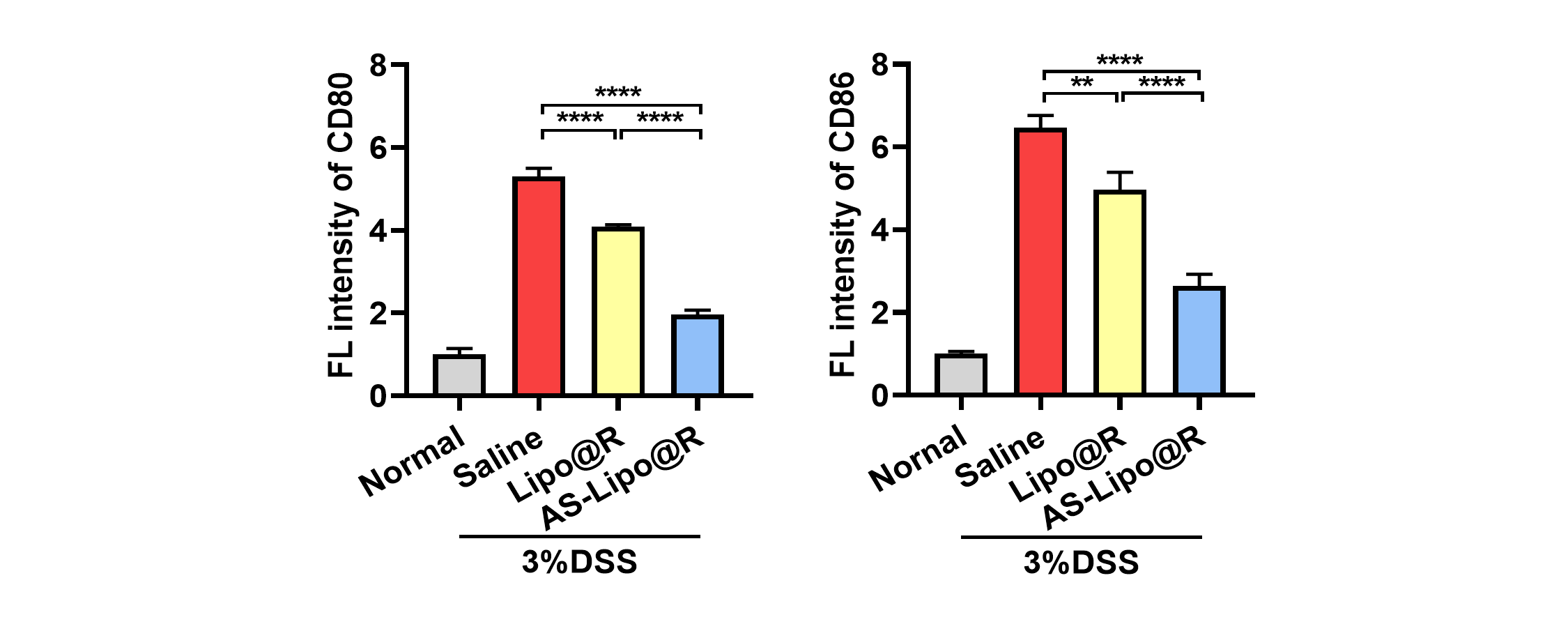
 Fig. S19.** Quantitative analysis of M1 macrophage marker expression (CD80 and CD86) in colonic tissues. Quantification of fluorescence intensity of CD80 and CD86 was performed on colon sections to evaluate the expression of M1-associated macrophage markers in DSS-induced colitis (n = 3 mice per group). Fluorescence intensity was measured from randomly selected fields of colonic mucosa and normalized to the tissue area. Data are presented as mean ± SD. Statistical significance was determined using one-way ANOVA followed by Tukey’s post hoc test. *p < 0.05, **p < 0.01, ***p < 0.001, ****p < 0.0001.


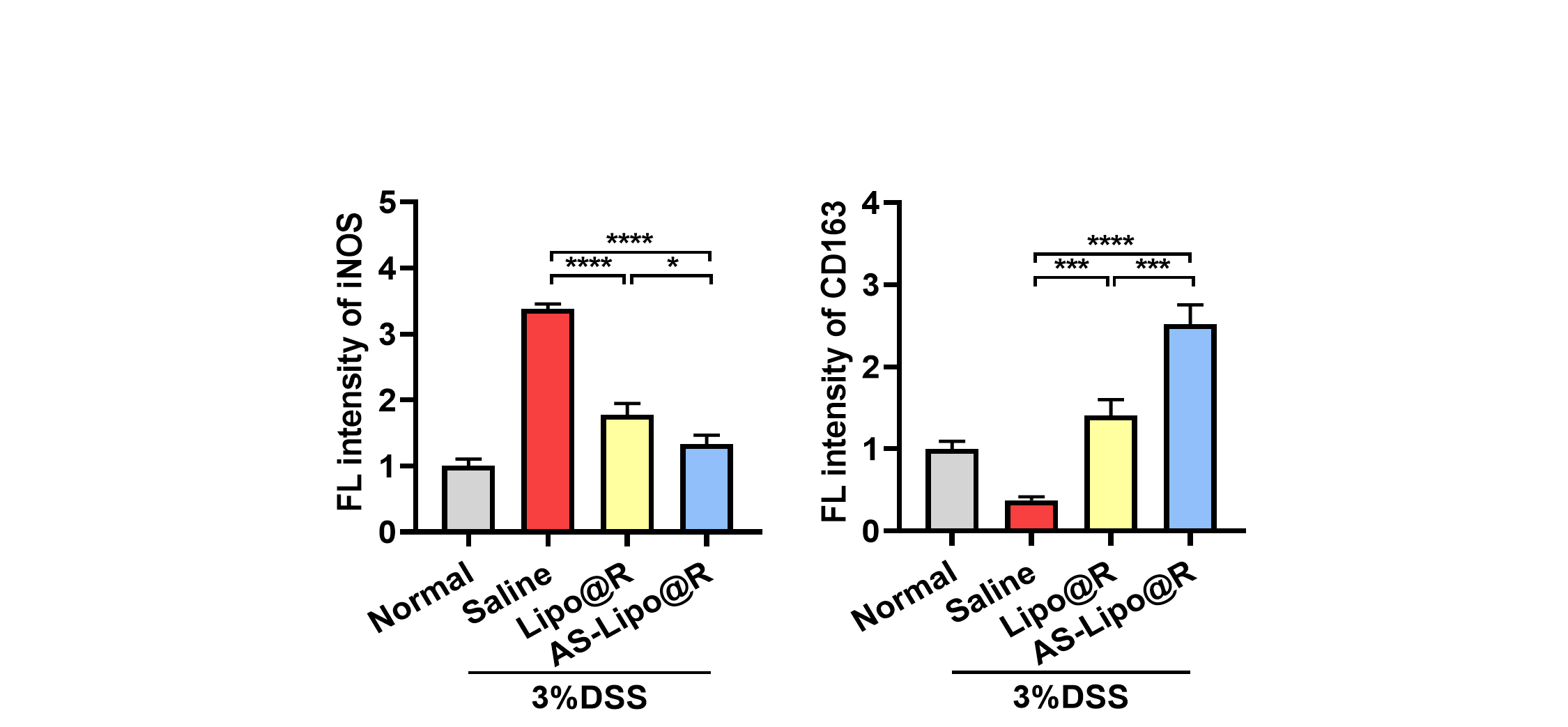
 **Fig. S20.** Quantitative analysis of iNOS and CD163 expression in colonic tissues. Fluorescence intensity of the M1-associated marker iNOS and the M2-associated marker CD163 was quantified in colon sections from DSS-induced colitis mice to assess macrophage polarization (n = 3 mice per group). Data are presented as mean ± SD. Statistical significance was determined using one-way ANOVA followed by Tukey’s post hoc test. *p < 0.05, **p < 0.01, ***p < 0.001, ****p < 0.0001.
